# Combining Ability and Heterosis for Endosperm Carotenoids and Agronomic Traits in Tropical Maize Lines

**DOI:** 10.3389/fpls.2021.674089

**Published:** 2021-09-09

**Authors:** Girum Azmach, Melaku Gedil, Charles Spillane, Abebe Menkir

**Affiliations:** ^1^Maize Improvement Unit, International Institute of Tropical Agriculture, Ibadan, Nigeria; ^2^Genetics and Biotechnology Laboratory, Plant and AgriBiosciences Research Centre, Ryan Institute, National University of Ireland Galway, Galway, Ireland

**Keywords:** maize, carotenoid, provitamin A biofortification, combining ability, hybrid vigor (heterosis), G × E interaction

## Abstract

Provitamin A enrichment of staple crops through biofortification breeding is a powerful approach to mitigate the public health problem of vitamin A deficiency in developing countries. Twenty-four genetically diverse yellow and orange endosperm maize inbred lines with differing levels of provitamin A content were used for the analysis of their combining ability. Each inbred line was developed from crosses and backcrosses between temperate and tropical germplasm. The inbred lines were grouped into different sets according to their provitamin A levels and were then intercrossed in a factorial mating scheme to generate 80 different single-cross hybrids. The hybrids were evaluated in field trials across a range of agroecological zones in Nigeria. The effect of hybrids was significant on all the measured provitamin A and non-provitamin A carotenoids and agronomic traits. While the effect of genotype-by-environment (GxE) interaction was significant for almost all traits, it was a non-crossover-type interaction for carotenoid content. Partitioning of the variances associated with the carotenoid and agronomic traits into their respective components revealed the presence of significant positive and negative estimates of general combining ability (GCA) and specific combining ability (SCA) effects for both carotenoid content and agronomic traits. The preponderance of GCA effects indicates the importance of additive gene effects in the inheritance of carotenoid content. We found F1 hybrids displaying high parent heterosis for both provitamin A content and agronomic performance. Our study demonstrates that provitamin A biofortification can be effectively implemented in maize breeding programs without adverse effects on important agronomic traits, including grain yield.

## Introduction

Hidden hunger or micronutrient deficiency remains a serious global public health problem. Biofortification of staple crops can be used to reduce the micronutrient deficiency burden in poorer communities. Biofortification is the application of conventional breeding and modern biotechnology to develop staple food crops with increased concentrations of bioavailable micronutrients in the edible parts of crops (Nestel et al., 2006; Miller and Welch, 2013). Maize is one of the most important staple crops feeding hundreds of millions of people in the developing world. Although maize is naturally endowed with the ability to accumulate carotenoids in the endosperm, the widely cultivated maize that is used for human consumption is deficient in provitamin A (Ranum et al., 2014). A range of maize biofortification breeding initiatives have developed and used maize inbred lines with high level of endosperm provitamin A to develop hybrids (Azmach et al., 2013; Babu et al., 2013; Pixley et al., 2013).

Determining the mode of inheritance and heterotic pattern of carotenoid content and agronomic traits is important to harness the genetic potential of such inbred lines for optimizing heterosis in commercial hybrid maize cultivars with improved provitamin A content. Improved knowledge on general combining ability (GCA) and specific combining ability (SCA) effects on the target traits is critical for heterosis-based biofortication breeding. Hybrid performance depends on the mode of gene action controlling the target trait and its heritability, which are both influenced by the underpinning genetic variation. Maize breeders in International Institute of Tropical Agriculture (IITA) utilize two heterotic groupings for yellow/orange maize inbred lines, namely, A and B, classified primarily based on grain yields. Backcrosses involving the adapted yellow/orange tropical recipient lines and the provitamin A donor lines were formed with due consideration of the existing heterotic groups. Although the heterotic affinities of the provitamin A donor lines to the lines belonging to the two heterotic groups were unknown, the existing genetic variation in carotenoid composition and content in the yellow and orange maize inbred lines (Menkir et al., 2008; Azmach et al., 2013) provides an opportunity to elucidate the mode of inheritance of carotenoid content for more efficient exploitation of heterosis to increase provitamin A concentrations in hybrid maize breeding.

The carotenoid content in maize kernels has been a subject of inheritance studies since the early twentieth century (Hauge and Trost, 1928, 1930; Hauge, 1930; Ford, 2000). However, when compared to yield and agronomic trait studies, there have been few recent studies focusing on the combining ability of maize for maize endosperm carotenoids. Previous studies have demonstrated the importance of additive gene action in inheritance of maize endosperm carotenoids, but were not consistent with respect to SCA effects (Grogan et al., 1963; Egesel et al., 2003; Senete et al., 2011; Li et al., 2013; Suwarno et al., 2014). The significance of additive genetic effects has been demonstrated for some of the major genes (e.g., *PSY1, lcyE*, and *crtRB1*) in the carotenoid biosynthetic pathway (Fu et al., 2010; Yan et al., 2010). Considering the inconsistent effects of SCA and the relatively low (and less variable) concentrations of β-carotene and β-cryptoxanthin in the parental lines used in some studies (e.g., Egesel et al., 2003), additional studies that involve the first generation of maize inbred lines with diverse genetic backgrounds and a broad range of carotenoid composition and content are necessary to more fully elucidate the mode of inheritance of these micronutrients in maize.

F1 hybrids with high provitamin A content need to also have high grain yield potential along with desirable agronomic traits if they are to be attractive to seed producers and farmers. While maize is known for its strong manifestation of heterosis for grain yield and other important agronomic traits, heterosis effects for carotenoid content in maize endosperm have been observed in only a limited number of studies (Burt et al., 2011; Alfieri et al., 2014). The identification of inbred lines that generate F1 hybrids with heterosis for both agronomic traits and carotenoids is critical for harnessing heterosis effects for biofortification breeding. Hence, it is necessary to evaluate provitamin A-biofortified maize inbred lines and their F1 hybrid progenies for both carotenoid composition and agronomic traits. Our objectives were to (i) determine the mode of inheritance of maize endosperm carotenoids and grain yield, including other key agronomic traits in a set of parental inbred lines developed by IITA, and (ii) assess their heterotic effects for carotenoid content.

## Materials and Methods

### Plant Materials and Crossing Scheme

Twenty-four yellow to orange endosperm maize inbred lines developed through biparental crosses and backcrosses involving tropical and temperate germplasm were used (Supplementary Table 1). These inbred lines were crossed in a factorial mating scheme (Hallauer et al., 2010) at IITA's main research station at Ibadan, Nigeria (7°29′11.99″N, 3°54′2.88″E, altitude 190 m) during two dry seasons (from December 2010 to April 2011 and December 2011 to April 2012). The parental inbred lines were divided into six different groups each containing four inbred lines to generate five sets of factorial crosses. The inbred lines were separated into different groups based on similarities in their heterotic affinities and carotenoid content determined in previous studies (Menkir et al., 2008; Azmach et al., 2013) to generate the five sets of crosses. Thus, each set of crosses involved inbred lines with different genetic backgrounds to maximize combining ability and heterosis. Generally, all the lines served as both male in one set and as a female parent in another set. The mating groups were crossed using the following arrangement: set 1: G-I × G-II, set 2: G-III × G-I, set 3: G-II × G-IV, set 4: G-V × G-III, and set 5: G-VI × G-V. This crossing scheme resulted in 80 F1 hybrids.

### Field Trial

The 80 F1 hybrids along with a commercial F1 hybrid check were arranged in a 9 × 9 lattice design and planted at four testing sites in Nigeria in 2012 and at two testing sites in 2013, making six agro-environments. The testing sites were Ikenne 3o42′E, 6o54′N, altitude 30 m; Saminaka 8o39′E, 10o34′N, altitude 760 m; Bagauda 8o19′E, 12o01′N, altitude 520 m; and Zaria 7o45′E, 11o8′N, altitude 622 m. The parental inbred lines were also tested along with the F1 hybrid trial separately in a 4 × 7 alpha lattice design at the Zaria and Saminaka field sites in both 2012 and 2013. Both F1 hybrid and inbred line trials were planted in two replications, where each entry was planted in a 5-m-long single row plot with a plant spacing of 75 cm between rows and 25 cm within a row. Two seeds per hill were planted but later thinned out to maintain one plant. The trials received all the agronomic management practices recommended for the respective field-testing site.

### Data Recording

The data were recorded on a plot basis. Agronomic data were collected from all the six environments (Ikenne, Saminaka, Bagauda and Zaria in 2012, plus Zaria and Saminaka in 2013), whereas carotenoid data were collected from the four environments (Zaria and Saminaka in both 2012 and 2013). Flowering dates were recorded based on the number of days at which 50% of the plants within a plot had set visible silks (as silking date) and started shedding sufficient pollen (as anthesis date). Ear and plant heights were measured in centimeters (cm) from the base of the plant to the attachment line of the lower ear and the first tassel branch, respectively. Ear aspect was scored on a 1 to 5 scale, where 1 represented the clean, well-filled, uniform, and larger ears, while 5 represented the diseased, poorly filled, variable, and smaller ears. Plant aspect was also scored on a 1–5 scale, where 1 represented the uniform, clean, vigorous, and good overall phenotypic appeal, while 5 represented the weak, diseased, and poor overall phenotypic appeal. Shelled grain weight of each F1 hybrid was measured in kilograms (kg) per plot, and moisture content of the representative sample grain was measured for each shelled plot using a portable moisture tester. The shelled grain weight per plot in kilograms was converted to grain yield in metric tons per hectare (t/ha) by first adjusting for 12.5% w/v moisture content. Grain yield was not recorded for the inbred line trial, since several plants in each plot were self-pollinated.

### Carotenoid Analysis

Five to six representative plants of each F1 hybrid and all typical plants of each inbred line were self-pollinated to produce seed samples for carotenoid analysis, from the field trials planted at Zaria and Saminaka during both 2012 and 2013 (i.e., four environments). Shelled seeds from self-pollinated ears were dried further at ambient temperature avoiding exposure to direct sunlight. Samples of about 100 seeds were drawn at random from each inbred line and F1 hybrid and subjected to carotenoid analysis.

Carotenoids were analyzed at the University of Wisconsin. Carotenoid extraction from maize grain samples was performed using the method of Howe and Tanumihardjo (2006). The equipment used for carotenoid separation and quantification was high-performance liquid chromatography (HPLC) system (Waters Corporation, Milford, MA). Details of the procedures for the analysis were described in the study by Azmach et al. (2018). The levels of α-carotene, β-carotene (cis and trans isomers), β-cryptoxanthin, lutein, and zeaxanthin were quantified in μg/g dry weight (DW). Total carotenoid was calculated as the sum of the concentrations of α-carotene, lutein, β-carotene, β-cryptoxanthin, and zeaxanthin. Provitamin A was calculated by adding the concentrations of β-carotene, and half of each of β-cryptoxanthin and α-carotene concentrations, since β-cryptoxanthin and α-carotene concentrations can provide only one molecule of retinol each as opposed to β-carotene, which can be converted to two molecules of retinol (US Institute of Medicine, 2001).

### Statistical Analysis

Mixed-model analysis using PROC MIXED procedure of SAS® software (SAS Institute, 2012) was employed for ANOVA among the F1 hybrids and the inbred line parents, and to obtain least-square means for each of the carotenoid traits and agronomic traits on a single and across environment basis after correcting for block effects. For traits with significant genotype-by-environment interaction (G×E), Spearman's rank correlation coefficients were calculated between environments in the PROC CORR procedure of SAS® software to determine whether the significant interactions of the F1 hybrids and parental inbred lines with the environment were of cross-over types.

The variability among the F1 hybrids within the mating sets was further analyzed using the data for the crosses formed through the factorial mating in our study (i.e., excluding the data for the check). This provided mean squares for females, males, and female-by-male interactions, including their interactions with the environments in the PROC GLM procedure of SAS® software. The linear mixed model used for the genetic analysis was:

$$\begin{array}{l} \text{Y}=\text{μ}+\text{El}+\text{Bk}\left(\text{l}\right)+\text{Sm}\left(\text{l}\right)+\text{ESl}\left(\text{m}\right)+\text{Fi}\left(\text{m}\right)+\text{EFi}\left(\text{m}\right)\\ \;+\text{Mj}\left(\text{m}\right)+\text{EMj}\left(\text{m}\right)+\text{FMij}\left(\text{m}\right)+\text{EFMij}\left(\text{m}\right)+\text{Error} \end{array}$$

where Y is the observed value from each experimental unit; μ is the grand mean; El is the effect of the lth environment; Bk(l) is the effect of the kth block nested within replication within each environment; Sm(l) is an mth set or mating group effect in lth environment; ESl(m) is the interaction effects between sets and the environment; Fi(m) is the main effect of the ith female within a set; EFi(m) is the interaction between females effect and the environment; Mj(m) is the main effect of jth male within the set; EMj(m) is the interaction between males effects and the environment; FMij(m) is the interaction effects of the cross between ith female and jth male; EFMijl(m) is the interaction between the SCA effects and the environment; and Error is the pooled residual effect.

In the mixed model, environments, replications within environment, and blocks within replication were considered as random effects, while mating sets, inbred lines (females and males), and hybrids were considered as fixed effects. GCA effects were inferred from variances of the main effects of males and females, while SCA effects were inferred from that of female-by-male interactions (Hallauer et al., 2010).

Family means-based heritability (H) estimates and the associated standard errors were calculated based on the method and code for SAS software provided by Holland et al. (2003), this time, assuming the genotypes were random samples representing a reference population.

$$\begin{array}{l} \text{H}=\frac{\text{V}\left(\text{G}\right)}{\text{V}\left(\text{G}\right)+\frac{\text{V}\left(\text{GE}\right)}{\text{e}}+\frac{\text{V}\left(\text{E }\right)}{\text{er}}} \end{array}$$

where V(G) is the variance due to genetic effect, V(GE) is the variance due to genotype-by-environment interaction, V(E) is the variance due to random errors, e is the number of environments, and r is the number of replications per environment.

Simple correlation coefficients were also calculated among carotenoids and agronomic traits. Correlation analyses were performed using PROC CORR of SAS software (SAS Institute, 2012).

Mid- and high parent heterosis and their significance were calculated as follows, according to the formula indicated in Hallauer et al. (2010) and Khorzoght et al. (2010).

$$\begin{array}{l} \text{mpH}=\frac{{\text{F}}_{1}-\text{mp}}{\text{mp}}\times 100,\text{ bpH}=\frac{{\text{F}}_{1}-\text{bp}}{\text{bp}}\times 100 \end{array}$$

where mpH is the mid-parent heterosis, bpH is the best parent heterosis, F1 is the hybrid value, and mp is an average value of the corresponding parents for the hybrid.

The significance of each hybrid value with respect to the corresponding mp and bp values was evaluated using t-test (Khorzoght et al., 2010):

$$\begin{array}{l} \text{Tmp}=\frac{{\text{F}}_{1}-\text{mp}}{\sqrt{\frac{3}{2\text{r}}\text{EMS}}},\text{ Tbp}=\frac{{\text{F}}_{1}-\text{bp}}{\sqrt{\frac{2}{\text{r}}\text{EMS}}} \end{array}$$

where Tmp is the calculated *t*-value for F1 to mp comparison and Tbp is for that of F1 to bp comparison, r is the number of repeats, and EMS is the mean square of error. The probability associated with the *t*-test was calculated using Microsoft Excel®.

## Results

### High Levels of Carotenoid Variability in the Seed Endosperm of Parental Inbred Lines and F1 Hybrids

The analysis of variance showed that both the inbred lines and F1 hybrids displayed significant variability in the carotenoid content of the seed endosperm (Table 1; Supplementary Table 2). The genetic variation among parents and F1 hybrids represented the largest proportion of the total variance for all the measured traits, followed by the variation due to environments, and F1 hybrid-by-environment interaction. Spearman's rank correlation analysis showed significant (*p* < 0.0001) positive pairwise correlations between pairs of environments for the concentration of each carotenoid, indicating that the environment-by-F1 hybrid interaction for carotenoids was not of the cross-over type, which means that genotypes did not differ in their relative carotenoid content with environmental changes. The strong impact of genetic effects on carotenoid variability was further confirmed by our estimates of heritability, which ranged from 57% for α-carotene to 95% for lutein in the parent trial, and 57% for α-carotene to 92% for β-cryptoxanthin in the F1 hybrid trial. The heritability estimates for provitamin A in both inbred line and F1 hybrid trials were 81%.

**Table 1:** Summary mean squares of combined ANOVA across environments for carotenoid contents of 80 maize F1 hybrids involving 24 inbred lines plus one commercial hybrid check.

**Source**	**DF**	**Lutein**	**Zeaxanthin**	**α-Carotene**	**β-Cryptoxanthin**	**β-Carotene**	**Provitamin A**	**Total carotenoid**
Envt	3	367.84^**^	413^**^	1.04^**^	26.72^**^	132.64^**^	183.63^**^	1,317.76^**^
Rep (Envt)	4	3	4.41	0.03	0.2	0.28	0.39	9.51
Block (Envt*rep)	64	3.33^*^	11.9^**^	0.02^**^	0.31^**^	0.75^**^	1.21^**^	27.12^**^
Hybrid	80	22.72^**^	62.51^**^	0.05^**^	3.61^**^	5.98^**^	7.15^**^	78.25^**^
Envt*Hybrid	240	3.23^**^	6.35	0.02^**^	0.34^**^	1.41^**^	1.63^**^	15.54^*^
Set	4	56.11^**^	274.94^**^	0.31^**^	41.11^**^	100.07^**^	119.4^**^	366.52^**^
Envt*set	12	7.63^**^	6.36	0.06^**^	1.53^**^	18.39^**^	18.82^**^	39.19^**^
Female (set)	15	72.1^**^	144.23^**^	0.08^**^	5.01^**^	5.47^**^	7.95^**^	145.02^**^
Male (set)	15	37.91^**^	131.4^**^	0.07^**^	7.5^**^	5.4^**^	7.17^**^	146.04^**^
Female*Male (Set)	45	5.31^**^	6.46	0.01	0.41^**^	0.64	0.98^*^	17.06
Envt*Female (Set)	45	3.67^*^	7.05	0.04^**^	0.44^**^	0.91^**^	1.04^*^	15.79
Envt*Male (Set)	45	4.27^**^	6.27	0.03^**^	0.51^**^	1.53^**^	1.96^**^	19.1
Envt*Female*Male (Set)	135	3.14	7.29	0.02^*^	0.2	0.39	0.61	18.2
Error	294	2.23	5.37	0.01	0.16	0.37	0.54	11.95
Corrected total SS	633	4,910.28	10,573.79	18.29	600.57	1,549.38	1,985.17	19,835.85
R^2^%		0.85	0.82	0.78	0.91	0.91	0.90	0.78
CV%		27.33	24.74	36.40	20.46	22.20	19.34	18.41
Grand mean		5.79	10.41	0.32	2.14	3.06	4.29	21.06
GCAf%		57	49	40	36	43	44	42
GCAm%		30	44	37	55	42	40	43
SCA%		13	7	23	9	15	16	15
H ± SE		0.87 ± 0.02	0.91 ± 0.02	0.92 ± 0.01	0.57 ± 0.08	0.79 ± 0.08	0.81 ± 0.04	0.81 ± 0.04

* *Significant at P < 0.05*,

** *significant at p < 0.01; Envt, Environment; Rep, Replication; DF, Degrees of freedom; GCAm%, GCA male proportion; GCAf%, GCA female proportion; H, Heritability; SE, Standard error*.

**Figure 1:**
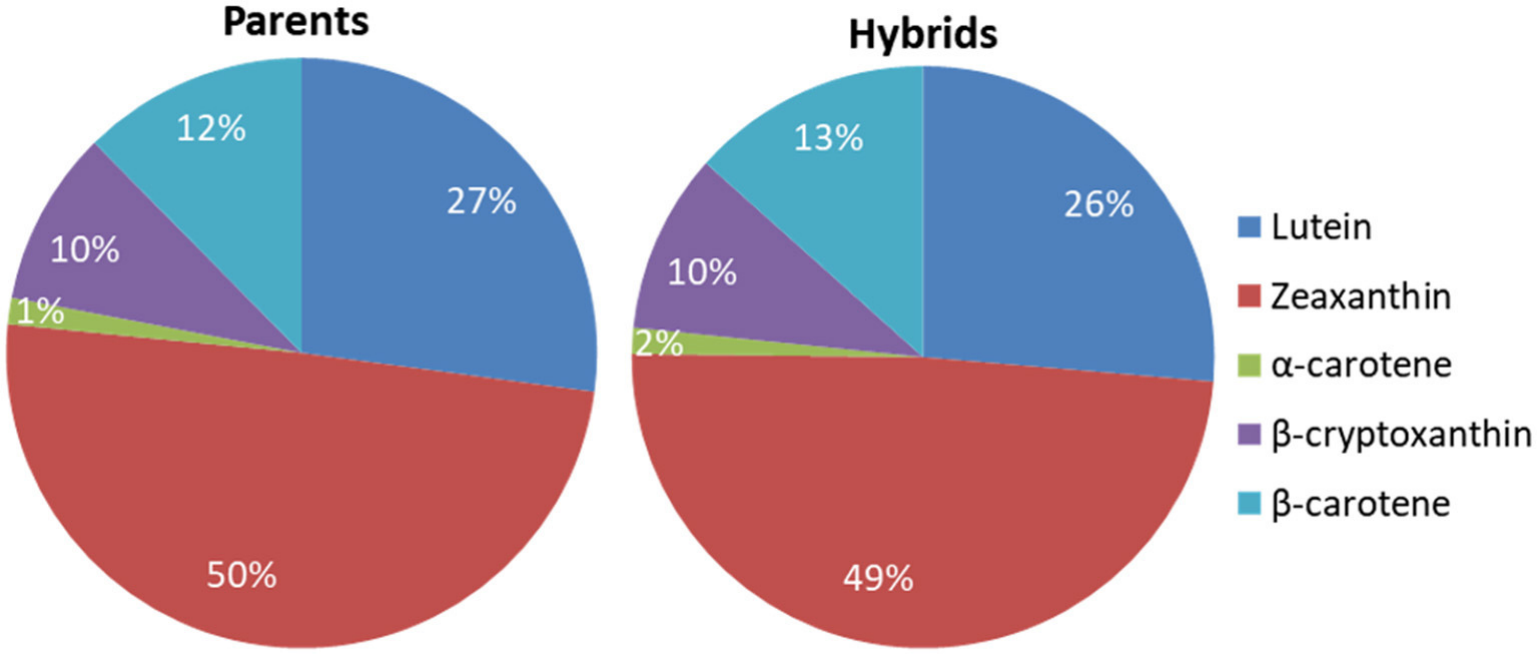
Proportions of the carotenoids in the endosperm of the parental inbred lines and the F1 hybrids averaged across environments.

**Figure 2:**
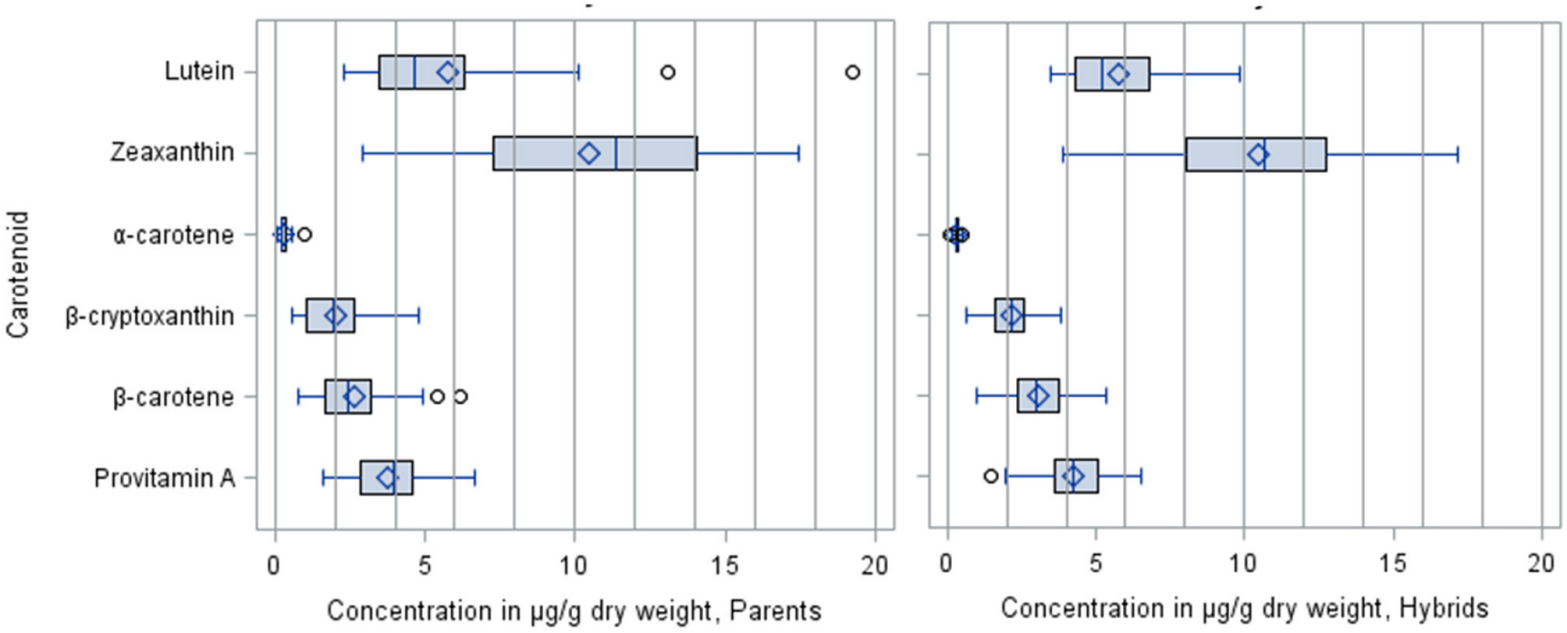
Distribution of carotenoid levels (μg/g) in the parental inbred lines and the F1 hybrids.

The mean carotenoid concentrations averaged over the environments are presented in Supplementary Tables 3, 4 for the inbred line and the F1 hybrid trials, respectively. The average proportions and distribution of the carotenoids in the inbred line and F1 hybrid trials were similar (Figures 1, 2). The distribution of all carotenoids measured in both the parents and F1 hybrids was broad, except for α-carotene (Figure 2). The average concentrations of the carotenoids in the inbred line trial were 5.53 μg/g for lutein, 10.29 μg/g for zeaxanthin, 2.01 μg/g for β-cryptoxanthin, and 2.49 μg/g for β-carotene, whereas, in the F1 hybrid trial, they were 5.79 μg/g for lutein 10.47 μg/g for zeaxanthin, 2.14 μg/g for β-cryptoxanthin, and 3.04 μg/g for β-carotene. The mean provitamin A content in the inbred line trial varied from 1.68 to 6.65 μg/g, whereas in the F1 hybrids, it varied from 1.44 to 6.53. The male and female parental lines of a commercial F1 hybrid check (Oba super 2) accumulated 3.08 μg/g and 2.72 μg/g provitamin A in their endosperm, respectively.

The set 2 hybrids (GIII × GI) displayed the highest mean concentration for zeaxanthin (8.58 μg/g), β-cryptoxanthin (2.76 μg/g), and total carotenoids (23.54 μg/g) (Table 2). The mean β-carotene (4.16 μg/g) and provitamin A (5.17 μg/g) concentrations of the F1 hybrids in set 1 (G-I × G-II) were found to be the highest, though they did not differ statistically from those of sets 2 and 3 for β-carotene, or those of sets 2, 3, and 4 for provitamin A. The F1 hybrids in set 5 (GVI × GV) displayed the lowest provitamin A carotenoid concentrations. This set displayed the highest average lutein concentration (6.92 μg/g).

**Table 2:** Mean carotenoid concentrations (μg/g) for the five mating sets.

**Set**	**Lutein**	**Zeaxanthin**	**α-Carotene**	**β-Cryptoxanthin**	**β-Carotene**	**Provitamin A**	**Total carotenoid**
1. G-I × G-II	5.41_B_	8.58_C_	0.37_A_	1.66_C_	4.16_A_	5.17_A_	19.6_B_
2. G-III × G-I	5.94_AB_	12.04_A_	0.37_A_	2.76_A_	3.4_AB_	4.88_A_	23.54_A_
3. G-II × x-IV	5.06_B_	10.87_AB_	0.32_AB_	2.18_B_	3.32_AB_	4.65_A_	21.14_B_
4. G-V × G-III	5.69_B_	11.45_A_	0.29_AB_	2.63_AB_	2.41_B_	3.87_AB_	21.67_AB_
5. G-VI × G-V	6.92_A_	9.2_BC_	0.27_B_	1.49_C_	1.97_B_	2.85_B_	19.43_B_
SE	0.81	0.89	0.04	0.23	0.57	0.64	1.53

*Carotenoid means within a column followed by similar letter subscript were not significantly different at P < 0.01; SE, Standard error; see section Materials and Methods for the description of sets. G, groups*.

### Combining Ability for Carotenoids

The female and male main effects (GCA) and interaction of each with the environment were highly significant for most of the measured carotenoids (Table 1). Although the female-by-male interaction (SCA) had significant effects on lutein, β-cryptoxanthin and provitamin A, the contribution of SCA to the total variation in hybrids was small, ranging from 7% to 16%. The interaction of SCA with the environment was not significant for almost all of the carotenoids.

#### General Combining Ability Effects for Carotenoids

The inbred lines showed significant (*P* < 0.01 and *P* < 0.05) positive and negative GCA effects for the carotenoids (Table 3), though the magnitudes fell largely between zero and one. The patterns of GCA effects seemed to be generally aligned with the groupings of the inbred lines. Inbred lines assigned to group I (inbred lines 1–4) showed significant positive male and female GCA effects for β-carotene and provitamin A content. Most of the group I inbred lines had negative male and female GCA effects for lutein, zeaxanthin, and β-cryptoxanthin. Inbred lines in group II (inbred lines 5 to 8) had positive female GCA effect for β-cryptoxanthin, and positive female and male GCA effects for β-carotene and provitamin A content, except inbred line 5 which showed a negative and significant female GCA effect for β-carotene and provitamin A. The inbred lines in group II also showed large positive female GCA effects for zeaxanthin and total carotenoids. However, inbred line 6 had positive GCA effects for all carotenoids excluding lutein. The inbred lines of group III (inbred lines 9 to 12) had variable GCA effects, while group IV inbred lines (inbred lines 13–16) showed largely positive male GCA effects for all carotenoids except lutein. The group V inbred lines showed negative GCA effects for most of the carotenoids. Similarly, inbred lines included in group VI (inbred lines 21–24), showed negative GCA effects for most of the measured carotenoids. The inbred lines 1, 4, 5, and 9 displayed relatively larger positive GCA effects (magnitude of 1.1–1.9) for provitamin A content when mated either as males or as females.

**Table 3:** Estimates of GCA effects for endosperm carotenoid content in 24 parental maize inbred lines used to generate 80 F1 hybrids.

**Parent**	**Group**	**Lutein**		**Zeaxanthin**		**β-Cryptoxanthin**		**β-Carotene**		**Provitamin A**		**Total carotenoid**	
		**Female**	**Male**	**Female**	**Male**	**Female**	**Male**	**Female**	**Male**	**Female**	**Male**	**Female**	**Male**
1	I	−0.82^**^	−0.25	−2.07^**^	−0.75^**^	−0.55^**^	−0.06	1.1^**^	0.44^**^	1.1^**^	0.44^**^	−2.19^**^	−0.61
2	I	−1.63^**^	−0.95^**^	−2.56^**^	1.71^**^	−0.43^**^	−0.46^**^	0.72^**^	0.33^**^	0.72^**^	0.33^**^	−3.81^**^	0.74^*^
3	I	−0.78^**^	−0.97^**^	0.84^**^	−0.2	0.08	0.62^**^	0.95^**^	0.6^**^	0.95^**^	0.6^**^	0.98^**^	−0.05
4	I	1.67^**^	−0.81^**^	−3.6^**^	1^**^	−1.04^**^	0.03	1.67^**^	0.02	1.67^**^	0.02	−0.9^**^	0.17
5	II	0.44^**^	−1.16^**^	−0.63^**^	−2.2^**^	0.25^**^	−0.63^**^	−0.32^**^	1.99^**^	−0.32^**^	1.99^**^	−0.33	−1.82^**^
6	II	−0.61^**^	−0.65^**^	2.36^**^	3.77^**^	0.49^**^	0.31^**^	0.42^**^	0.59^**^	0.42^**^	0.59^**^	2.58^**^	3.94^**^
7	II	1.36^**^	0.09	2.04^**^	−4.14^**^	0.69^**^	−0.98^**^	0.54^**^	0.97^**^	0.54^**^	0.97^**^	4.42^**^	−3.77^**^
8	II	−0.63^**^	0.16	2.67^**^	−4.83^**^	1.05^**^	−0.63^**^	0.42^**^	0.9^**^	0.42^**^	0.9^**^	3.21^**^	−4.26^**^
9	III	−1.14^**^	−0.04	1.65^**^	−1.16^**^	0.17^**^	0.1	1.11^**^	−1.12^**^	1.11^**^	−1.12^**^	1.66^**^	−2.34^**^
10	III	−1.29^**^	−1.09^**^	4.41^**^	1.65^**^	0.65^**^	0.67^**^	−0.08	−0.5^**^	−0.08	−0.5^**^	3.57^**^	0.46
11	III	−0.85^**^	1.87^**^	−1.58^**^	1.91^**^	−0.49^**^	0.28^**^	0.13	−0.48^**^	0.13	−0.48^**^	−2.65^**^	3.62^**^
12	III	0.28	−1.19^**^	−2.72^**^	1.7^**^	−0.18^**^	0.9^**^	0.23^**^	−0.46^**^	0.23^**^	−0.46^**^	−2.33^**^	0.62
13	IV		−0.62^**^		2.24^**^		0.71^**^		0.45^**^		0.45^**^		2.61^**^
14	IV		−1.3^**^		0.48^*^		0.69^**^		−0.24^*^		−0.24^*^		−0.61
15	IV		−0.34^*^		3.22^**^		1.4^**^		0.23^*^		0.23		4.09^**^
16	IV		2.82^**^		0.52^*^		−0.33^**^		0.62^**^		0.62^**^		3.77^**^
17	V	−0.9^**^	0.67^**^	1.68^**^	−0.05	0.38^**^	−0.64^**^	−0.83^**^	−1.11^**^	−0.83^**^	−1.11^**^	0.06	−0.95^*^
18	V	0.44^**^	1.13^**^	2.98^**^	−0.38	0.49^**^	−0.61^**^	−0.7^**^	−1.21^**^	−0.7^**^	−1.21^**^	3.04^**^	−0.79^*^
19	V	−0.22	1.45^**^	−0.27	−2.64^**^	0.26^**^	−0.91^**^	−0.91^**^	−1.53^**^	−0.91^**^	−1.53^**^	−1.22^**^	−3.39^**^
20	V	0.22	1.2^**^	−0.29	−1.83^**^	0.82^**^	−0.46^**^	−0.12	−0.48^**^	−0.12	−0.48^**^	0.49	−1.45^**^
21	VI	3.56^**^		−3.36^**^		−1.2^**^		−1.49^**^		−1.49^**^		−2.21^**^	
22	VI	−1.28^**^		−0.93^**^		−0.3^**^		−0.7^**^		−0.7^**^		−3.04^**^	
23	VI	3.38^**^		−1.63^**^		−0.7^**^		−0.76^**^		−0.76^**^		0.65^*^	
24	VI	−1.21^**^		1.02^**^		−0.42^**^		−1.38^**^		−1.38^**^		−1.98^**^	
**SE**		0.15	0.16	0.20	0.19	0.20	0.19	0.05	0.06	0.07	0.10	0.08	0.11
**GM**		5.80	5.80	10.43	10.43	2.14	2.14	3.05	3.05	3.05	3.05	21.08	21.08

* *Significant at P < 0.05*,

** *significant at p < 0.01. SE, Standard error; GM, Grand mean*.

Overall, our results demonstrate that group VI inbred lines as females and group V inbred lines as males were poor combiners for all of the carotenoids, except for lutein. Likewise, group V inbred lines as females and group III inbred lines as males were poor combiners for carotenoid content, but not for β-cryptoxanthin. The inbred lines included in group I and II were generally good combiners as both female and male parents, especially for β-carotene and total provitamin A carotenoids. The largest GCA effect on β-carotene and provitamin A was recorded for inbred line 5 as a male. This inbred line accumulated the highest level of provitamin A (6.65 μg/g) but had low total carotenoid content (12.13 μg/g). The largest GCA effects on zeaxanthin and total carotenoids were recorded for inbred line 6 as both female and male, and for inbred lines 7, 8, 9, 10 as female parents. The total carotenoid contents of these inbred lines ranged from 13.7 μg/g for inbred line 8 to 24.63 μg/g for inbred line 6.

#### Specific Combining Ability Effects for Carotenoids

Many of the crosses showed significant (*P* < 0.01 or *P* < 0.05) negative and positive SCA effects on carotenoids (Supplementary Table 5). Crosses of set 1 (GI × GII) had significant positive SCA effects on β-cryptoxanthin, while a number of the crosses in this set had large positive SCA effects on zeaxanthin. On the other hand, set 1 crosses displayed significant negative SCA effects on β-carotene and provitamin A. Apart from the negative SCA effects of set 2 crosses (G III × G I) for β-cryptoxanthin, the SCA effects for most of the crosses in sets 2 and 3 were not significant. All of the crosses in sets 4 and 5 (G V × G III and G VI × G V) displayed positive SCA effects for individual and total provitamin A carotenoids, except for four crosses that showed negative SCA effects for β-cryptoxanthin.

### Agronomic Performance of Inbred Line Parents and F1 Hybrids

Both the F1 hybrids and the inbred line parents exhibited significant differences in all the agronomic traits evaluated (Table 4; Supplementary Table 6). Hybrid means averaged across environments ranged from 56 to 63 days for days to anthesis (AD), from 0.66 to 5.87 t/ha for grain yield (GY), from 2.3 to 4.36 for ear aspect (EA), and from 2.45 to 4.34 for plant aspect (PA) (Supplementary Table 7). The F1 hybrids 24 (12 × 3), 32 (12 × 1) and 52 (20 × 12) had the highest grain yield (5.7 to 5.9 t/ha), but were not significantly different from circa half of the F1 hybrids, including the commercial check. The ear aspect and plant aspect scores of these top-yielding F1 hybrids were acceptable (< score of 3), while their provitamin A contents were higher than 5 μg/g, which were not significantly different from the highest provitamin A concentration in the F1 hybrid trial (6.5 μg/g).

**Table 4:** Summary of mean squares of combined ANOVA across environment for agronomic traits of 80 F1 hybrid maize progenies plus one commercial hybrid check.

**Source**	**DF**	**SD**	**AD**	**PH**	**EH**	**GY**	**EA**	**PA**
Envt	5	2,045.94^**^	1,958.78^**^	77,377.7^**^	38,520.3^**^	1,157.84^**^	16.18^**^	9.08^**^
Rep (Envt)	6	13.09^**^	12.05^**^	926.49^**^	398.34^**^	4.39^**^	0.35	1.19^**^
Block (Envt*rep)	96	7.59^**^	4.84^**^	706.71^**^	354.61^**^	1.52^**^	0.4^**^	0.37^**^
Hybrid	80	30.9^**^	23.71^**^	1,371.78^**^	889.44^**^	4.63^**^	1.2^**^	1.16^**^
Envt*Hybrid	400	3.08^**^	1.82^**^	229.64^**^	105.67	1.05^**^	0.35^**^	0.35^**^
Set	4	66.36^**^	68.64^**^	1,917.41^**^	1,939.31^**^	4.39^**^	2.83^**^	1.42^**^
Envt*set	20	5.69^*^	2.72	287.73	72.98	2.02^**^	0.87^**^	0.61^**^
Female (set)	15	108.67^**^	78.26^**^	2,380.92^**^	2,133.19^**^	7.07^**^	2.27^**^	2.1^**^
Male (set)	15	50.25^**^	39.13^**^	3,765.76^**^	2,426.06^**^	10.32^**^	1.02^**^	2.04^**^
Female*Male (set)	45	8.92^**^	6.18^**^	1,009.79^**^	356.99^**^	2.91^**^	0.86^**^	0.75^**^
Envt*Female (set)	75	7.65^**^	3.4^**^	358.59	174.13	1.47^**^	0.62^**^	0.57^**^
Envt*Male (set)	75	4.5^*^	3^*^	343.62	181.58	1.93^**^	0.51^**^	0.63^**^
Envt*Female*Male (set)	225	2.74	1.96	265.75	113.88	0.99^*^	0.31	0.31^**^
Error	450	3.09	2.12	276.61	144.84	0.74	0.26	0.24
Corrected total SS	959	13,663.71	11,681.12	77,7988.00	40,6561.60	5,767.09	479.28	440.68
Grand Mean		60.30	58.53	192.83	92.00	4.57	3.03	3.09
*R*^2^%		91.59	93.20	84.04	84.00	95.20	75.32	75.89
CV%		2.91	2.49	8.62	13.09	18.87	16.89	15.70
GCAf%		59	50	26	38	26	27	33
GCAm%		27	27	41	43	40	40	32
SCA%		14	23	33	19	34	33	35
Heritability (H ± SE)		90 ± 2	92 ± 1	81 ± 3	87 ± 2	76 ± 4	62 ± 6	61 ± 6

* *Significant at P < 0.05;*

** *significant at p < 0.01; GCAm%, GCA male proportion; GCAf%, GCA female proportion; CV%, coefficient of variation in%; R2%, percentage of variation explained SE, Standard error; DF, degree of freedom; SD, no. of days to silking; AD, no. of days to anthesis; PH, plant height (in cm); EH, ear height (in cm); GY, grain yield (in t/ha); EA, ear aspect (1–5 score); PA, plant aspect (1–5 score); Envt, Environment; Rep, Replication; DF, Degrees of Freedom; R2%, percent explained variance; H, Heritability; SE, Standard Error*.

Female and male main effects were highly significant for all agronomic traits (**Table 6**). The effects of female-by-male interaction (SCA effects) were significant for all the traits. Rank correlation coefficients for the agronomic characters were non-significant, suggesting the presence of changes in ranking of the hybrids across environments (data not presented). The proportion of female GCA effects relative to the total variation for the F1 hybrids ranged from 26% for plant height to 59% for SD, and the proportion for male GCA effect ranged from 27% for AD to 43% for EA. The SCA component of variance ranged from 14% for SD to 35% for PA, which were higher than the SCA component of variance observed for carotenoids.

#### Effects of GCA for Agronomic Traits

The inbred lines displayed significant (*p* < 0.01 and 0.05) negative and positive GCA effects for all the agronomic traits (Table 5). Inbred lines with positive GCA effects were considered good combiners for GY, while inbred lines having negative GCA effects were considered good combiners for PA and EA. For AD, both positive and negative GCA effects can be desirable depending on preferences for early or late maturity. For the PH trait, shorter cultivars are typically preferred to minimize lodging. Inbred line 12 had positive GCA effects for GY and negative GCA effects for PA and EA as both male and female, making it the best combiner for the three important agronomic traits in the favorable direction (higher GY and lower PA and EA scores in the 1, good, to 5, bad, scale). In addition, inbred lines 19 and 20 had significant positive GCA effects as females and males for GY. Inbred lines 2, 5, 7, 13 and inbred lines 6, 21, 22, 23 displayed positive GCA effects for GY as males and females, respectively. Inbred lines 4 and 7 had negative female and male GCA effects for PA. Inbred lines 6 and 21 had negative GCA effects as females, whereas inbred lines 15 and 16 had negative GCA effects as males for the same trait. Inbred lines 6, 11, 20, and 21 displayed negative GCA effects for EA when used as females, and inbred lines 2, 3, and 18 displayed negative GCA effects for EA when used as males. Most of the inbred line recorded negative female and male GCA effects for AD and PH. Inbred line 10, 22, and 23 had large positive GCA effects for both AD and PH.

**Table 5:** Estimates of GCA effects for agronomic traits in 80 F1 maize hybrids produced through factorial mating of 24 inbred lines.

**Parent**	**Group**	**AD**		**PH**		**GY**		**PA**		**EA**	
		**Female**	**Male**	**Female**	**Male**	**Female**	**Male**	**Female**	**Male**	**Female**	**Male**
1	I	−0.55^**^	−0.81^**^	−4.93^**^	0.82	−0.39^**^	0.16	0.36^**^	0.13^*^	0.29^**^	−0.09
2	I	−0.96^**^	−0.6^**^	2.13	8.76^**^	−0.08	0.33^**^	0.24^**^	0.07	0.09	−0.09^*^
3	I	0.32^*^	−0.64^**^	−9.12^**^	10.53^**^	−0.72^**^	0.18	0.22^**^	0.01	0.27^**^	−0.2^**^
4	I	−0.35^*^	0.46^**^	6.51^**^	1.15	0.03	−0.46^**^	−0.32^**^	−0.11^*^	0.12^*^	0.04
5	II	−0.98^**^	−1.58^**^	−9.26^**^	3.68^**^	0.06	0.32^**^	0.02	0.03	0.25^**^	0.18^**^
6	II	−0.75^**^	1.38^**^	9.41^**^	−9.07^**^	0.57^**^	−1.82^**^	−0.19^**^	0.57^**^	−0.49^**^	0.58^**^
7	II	−1.7^**^	−1.72^**^	−8.74^**^	0.36	0	0.29^**^	−0.24^**^	−0.23^**^	0	−0.07
8	II	1.21^**^	0.37^**^	−7.66^**^	−0.39	−0.36^**^	0.06	0.18^**^	0.14^*^	0.36^**^	0.09
9	III	−0.46^**^	0.74^**^	1.64	−4.11^**^	−0.2^*^	−0.3^**^	0.08	−0.02	−0.06	0.03
10	III	1.57^**^	1.27^**^	14.11^**^	9.49^**^	−0.39^**^	0.06	0.24^**^	0.07	0.18^**^	0.08
11	III	−1.36^**^	−0.7^**^	−4.59^**^	−9.55^**^	0.03	0.2	0.06	−0.14^*^	−0.22^**^	−0.07
12	III	−1.33^**^	−0.38^**^	10.09^**^	−1.53	0.76^**^	0.34^**^	−0.28^**^	−0.24^**^	−0.25^**^	−0.23^**^
13	IV	–	−0.16	–	15.09^**^	–	0.31^**^	–	0.13^*^	–	0.02
14	IV	–	−1.01^**^	–	−14.64^**^	–	−0.02	–	0.07	–	0.04
15	IV	–	−1.33^**^	–	−9.78^**^	–	−0.17	–	−0.26^**^	–	0.03
16	IV	–	0.27^*^	–	−6.92^**^	–	0.16	–	−0.18^**^	–	0.04
17	V	0.71^**^	1.61^**^	1	6.87^**^	−0.47^**^	−0.25^*^	−0.09	−0.02	0.07	−0.06
18	V	1.08^**^	1.85^**^	−1.04	6.18^**^	0.04	−0.05	−0.25^**^	−0.27^**^	−0.01	−0.18^**^
19	V	−0.61^**^	0.64^**^	−6.3^**^	−8.62^**^	0.18^*^	0.35^**^	0.05	0.05	0.05	−0.09
20	V	−0.25	0.33^*^	0.64	1.68	0.53^**^	0.33^**^	−0.04	0.19^**^	−0.3^**^	−0.03
21	VI	−1.6^**^	–	−2.82^*^	–	0.6^**^	–	−0.23^**^	–	−0.32^**^	
22	VI	0.39^**^	–	2.86^*^	–	0.22^*^	–	0.01	–	−0.1	
23	VI	1.71^**^	–	9.48^**^	–	0.2^*^	–	0.17^**^	–	−0.06	
24	VI	3.92^**^	–	−3.41^**^	–	−0.63^**^	–	0.01	–	0.12^*^	
SE		0.13	0.12	1.19	1.17	0.83	0.85	0.08	0.10	0.05	0.05

* *Significant at P < 0.05*,

** *significant at p < 0.01, AD, no. of days to anthesis; PH, plant height (in cm); GY, grain yield (in t/ha); EA, ear aspect (1 to 5); PA, plant aspect (1–5)*.

#### Effects of SCA for Agronomic Traits

Several of the F1 hybrids showed significant SCA effects for important agronomic traits (Supplementary Table 8). The hybrids generated in set 1 and 5 displayed most of the significant SCA effects for agronomic traits considered. Hybrids of set 1 had both positive and negative SCA effects for all the measured traits, whereas hybrids in set 5 had only negative SCA effects for SD and AD dates. The best hybrids 7 (3 × 7) and 12 (4 × 6) had large positive SCA effects for PH and GY and negative SCA effects for PA, EA, SD, and AD.

Four high-yielding hybrids, namely, 24 (5.71 t/ha), 28 (5.42 t/ha), 32 (5.87 t/ha), and 52 (5.68 t/ha), had inbred line 12 as a common parent, which showed positive GCA effect for provitamin A when crossed as a female parent (Table 3) and for grain yield when crossed as a female and male parent (Table 5). Inbred line 12 also showed negative female and male GCA effects for PA and EA which is desirable. The other non-common parents of the high-yielding hybrids were also good combiners for provitamin A except inbred line 20. Hybrid 38 (6 × 15) had the best EA and PA, but its SCA estimates were negative for all carotenoids (Supplementary Table 5). However, its two parents (inbred line 6 and 15) were good combiners for individual and total provitamin A carotenoids (Table 3).

### Correlation and Regression Analyses of Agronomic and Carotenoid Traits

The hybrids displayed either significant positive or negative correlations between combinations of carotenoids, except for β-carotene with lutein, zeaxanthin, and β-cryptoxanthin (Table 6). In the inbred trial, α-carotene with lutein (*r* = 0.42, *p* < 0.05), α-carotene with β-carotene (*r* = 0.49, *p* < 0.05), and β-cryptoxanthin with zeaxanthin (*r* = 0.66, *p* < 0.01) were found to be significantly correlated. The strength and direction of correlation reflected the positions of the carotenoid biomolecules along the linear representation of the carotenoid biosynthesis pathway and the underlying enzymes involved in the bioconversions and synthesis of the different carotenoid molecules. The correlation between β-carotene and β-cryptoxanthin was not significant, whereas the correlation between α-carotene and β-carotene were strong and positive.

**Table 6:** Pearson's correlation coefficients between mean values of carotenoid concentrations in the parent (upper-half matrix) and F1 hybrid (lower-half matrix) maize trials.

	**Parents**						
	**Lut**	**Zea**	**αcar**	**βcryp**	**βcar**	**PVA**	**Tcar**
Lut	1	−0.2	0.42^*^	−0.19	−0.07	−0.11	0.55^**^
Zea	−0.28^*^	1	−0.22	0.63^**^	−0.37	−0.14	0.66^**^
αcar	0.45^**^	−0.3^*^	1	0.02	0.49*â	0.54^**^	0.27
βcryp	−0.28^*^	0.66^**^	0.11	1	−0.19	0.18	0.46^*^
βcar	−0.02	0.05	0.66^**^	0.14	1	0.93^**^	−0.1
PVA	−0.13	0.27^*^	0.64^**^	0.45^**^	0.94^**^	1	0.08
Tcar	0.24^*^	0.84^**^	0.44^**^	0.61^**^	0.28^*^	0.45^**^	1
	Hybrids						

* *Significant at P < 0.05*,

** *significant at P < 0.01; Lut, Lutein; zea, Zeaxanthin; αcar, α-carotene; βcryp, β-cryptoxanthin; βcar, β-carotene; PVA, Provitamin A; tcar, Total carotenoid*.

Correlations between the carotenoids and agronomic traits were in general not significant (Table 7). However, the correlation of α-carotene with each of the agronomic traits was significant and negative, except with ASI and PA, whereas the correlations of total carotenoid with SD, AD, PH, and GY were significant and positive. Beta-carotene and β-cryptoxanthin were not associated with GY.

**Table 7:** Pearson's coefficient of correlation between carotenoids and agronomic traits averaged across environments and regression of carotenoid content of the F1 hybrids on their mid-parent values.

	**Lut**	**Zea**	**αcar**	**βcryp**	**βcar**	**PVA**	**Tcar**
SD	0.06	0.17	−0.6^**^	−0.09	−0.12	0.17	0.41^**^
AD	0.06	0.17	−0.61^**^	−0.09	−0.12	0.16	0.41^**^
ASI	0.02	−0.04	0.05	0.06	0.09	0.01	−0.18
PH	0.01	0.11	−0.5^**^	−0.1	−0.12	0.28^*^	0.37^**^
EH	0.01	0.03	−0.39^**^	−0.08	−0.09	0.14	0.19
GY	−0.17	−0.17	−0.48^**^	−0.29^**^	−0.3^**^	0.23^*^	0.3^**^
EA	0.03	0.02	−0.29^**^	−0.01	0	0.12	0.03
PA	−0.09	−0.01	0.05	−0.08	0.1	0.06	−0.04
*r*	0.29^**^	0.39^**^	−0.04	0.39^**^	0.14	0.17	0.16
*R*^2^%	8.23	15.59	0.18	15.49	2.07	2.74	2.52

* *Significant at p < 0.01*,

** *significant at p < 0.05; Lut, Lutein; zea, Zeaxanthin; αcar, α-carotene; βcryp, β-cryptoxanthin; βcar, β-carotene; PVA, Provitamin A; tcar, Total carotenoid; SD, no. of days to silking; AD, no. of days to anthesis; PH, plant height (in cm); GY, grain yield (in t/ha); EA, ear aspect; PA, plant aspect; r, coefficient for regression of hybrids carotenoid content on mid-parent values; R^2^, percentage of variation in carotenoid content of the hybrids explained by that of the mid-parent values*.

The regression of the carotenoid content of the F1 hybrids on mid-parent values detected significant (*p* < 0.01) but weak associations for lutein (*r* = 0.29), zeaxanthin (*r* = 0.39), and β-cryptoxanthin (*r* = 0.39) (Table 7). The regression coefficients for β-carotene, provitamin A, and total carotenoids were non-significant.

### Heterosis Effects on Carotenoids and Agronomic Traits

Fifteen of the F1 hybrids displayed significant high parent heterosis (hpH%) (Table 8), while 42 of the F1 hybrids displayed positive mid-parent heterosis (mpH%) for at least one of the carotenoids (Supplementary Table 9). The largest number of hybrids with positive mpH% heterosis was observed for β-carotene (22 hybrids) followed by β-cryptoxanthin (21 hybrids), zeaxanthin (17 hybrids), and lutein (14 hybrids). Significant mid-parent heterosis effects were detected for β-carotene, provitamin A, and total carotenoids, varying from 15 to 55%. Hybrid 30 (10 × 1) exhibited mpH for all carotenoids and had provitamin A content of 5.3 μg/g, but was poor in its agronomic traits. Mid-parent heterosis reached up to 77% for zeaxanthin and 114% for lutein. Hybrids 22 (10 × 3) and 23 (11 × 3) displayed a significant positive high parent heterosis for both β-cryptoxanthin and β-carotene, ranging from 30 to 56% with mean provitamin A concentrations of 5.27 and 6.0 μg/g, respectively. Hybrid 12 (4 × 6), which showed favorable SCA effects for the most important agronomic traits GY, EA, and PA, expressed high parent heterosis of 32% for provitamin A (6.06 μg/g) and 37% for total carotenoid (25.58 μg/g). This hybrid was comparable to the best-performing hybrid in terms of its agronomic traits, having 4.9 t ha-1 for GY, and 2.6 for EA and PA. Hybrid 38 (6 × 15) also showed significant positive high parent heterosis for provitamin A (21%), high provitamin A concentration (5.32 μg/g), and good agronomic characters, 5.2 t/ha for GY, 2.3 for EA, and 2.5 for PA.

**Table 8:** High parent heterosis (hPH%) estimates, provitamin A content, and agronomic performances of 15 F1 hybrids, out of the 80 F1 hybrids generated from 24 inbred lines of maize in a factorial mating design.

**Hybrid**	**F**	**M**	**Set**	**High parent heterosis**						**PVA (ìg/g)**	**GY (t/ha)**	**EA**	**PA**
				**Lut**	**Zea**	**βcryp**	**βcar**	**PVA**	**Tcar**				
3	3	8	1	12.38	−42.77^***^	−15.77	31.89^**^	46.72^***^	−5.34	5.82	4.73	3.20	3.37
9	1	6	1	44.11^*^	−15.61	−19.83^*^	−5.26	1.4	−4.33	5.03	2.62	3.81	3.78
12	4	6	1	−21.14^*^	−15.95	−32.54^***^	31.82^**^	37.36^***^	7.91	6.06	4.89	2.59	2.58
13	1	5	1	112.22^**^	−22.74	−15.21	−20.29^**^	−12.01^*^	4.42	5.85	4.98	3.30	3.62
22	10	3	3	−11.71	6	29.84^**^	33.97^*^	32.9^**^	12.46	5.27	4.43	3.10	3.34
23	11	3	3	−21.08^*^	3.74	38.6^***^	55.63^***^	51.36^***^	3.93	6.00	4.65	2.77	2.96
27	11	2	3	−43.56^***^	−13.26	22.03^*^	16.68	19.46	−20.43^**^	4.72	4.90	2.83	3.30
30	10	1	3	14.97	21.67^*^	−2.89	−1.5	8.68	16.69	5.39	3.73	3.40	3.42
31	11	1	3	−31.07^**^	1.89	19.2^*^	−0.78	12.21	−6.37	5.57	4.74	2.88	3.18
38	6	15	2	6.63	−3.51	18.99^*^	20.78	21.3^*^	3.3	5.32	5.23	2.30	2.45
56	20	11	4	−10.55	−5.28	20.23^*^	16.36	19.15^*^	−4.54	4.98	4.79	2.89	3.06
57	17	10	4	−17.57	23.14^*^	26.81^*^	−1.66	6.35	4.63	4.22	3.93	3.43	3.29
58	18	10	4	0.89	27.86^*^	−4.62	4	0.07	11.33	3.97	4.54	3.16	3.07
79	23	17	5	−52.56^***^	4.57	47.66^*^	4.55	12.04	−22.51^**^	3.25	4.55	2.89	3.06
80	24	17	5	39.05	34.61^*^	3.6	23.49	29.36	37^**^	2.24	3.49	3.34	3.03

*F, Female Parent; M, Male Parent; Lut, Lutein; Zea, Zeaxanthin; βcryp, β-cryptoxanthin; βcar, β-carotene; PVA, Provitamin A; tcar, Total carotenoid. Only crosses with significant negative or positive heterosis for at least one of the carotenoids are shown*.

* *Significant at p < 0.05*,

** *significant at p < 0.01*,

*** *significant at p < 0.001*.

Some of the F1 hybrids displayed heterosis for multiple carotenoids. For example, F1 hybrid 30 showed a significant mid-parent heterosis for all carotenoids, while F1 hybrids 15, 57 and 58 showed significantly positive mid-parent heterosis for all β-branch carotenoids. Most F1 hybrids that displayed significantly positive heterosis for carotenoids in the β-branch did not show significantly positive heterosis for lutein. Similarly, most of the F1 hybrids that displayed significantly positive heterosis for at least one provitamin A carotenoid did not show significant heterosis for the non-provitamin A carotenoids zeaxanthin and lutein.

## Discussion

Analysis of combining ability is an important component of maize breeding to identify inbred lines with good breeding value for F1 hybrid and synthetic formation. Combining ability helps to better understand the mode of gene action controlling the trait of interest and devise breeding strategies toward improving the trait. In this study, the combining ability of 24 inbred lines for carotenoid and important agronomic traits was investigated. Both the parental inbred lines and their hybrids showed broad ranges of variation in carotenoids and agronomic traits. The magnitude of provitamin A levels observed in this study is comparable with results reported to date for tropical diverse yellow maize inbred lines (Menkir et al., 2008). While some combining ability studies have reported slightly lower levels of beta-carotene and provitamin A (Egesel et al., 2003; Senete et al., 2011; Li et al., 2013) than our study, such studies evaluated smaller numbers of F1 hybrids (e.g., 25–52) than the hybrids that were evaluated in our study. However, a recent combining ability study on maize carotenoid that used 21 inbred lines and 156 hybrids reported higher levels of provitamin A ranging from 4.4 to 18 μg/g (Suwarno et al., 2014).

Although significant interactions were detected between most of the individual carotenoids and the environment, the strong positive correlation of carotenoids' means across environments suggested the stable accumulation of each carotenoid trait despite environmental changes. The high heritability estimates detected for all carotenoid traits except α-carotene further corroborated the strong genetic effect on carotenoid content variability in both the parents and their hybrid progenies.

Zeaxanthin was the predominant carotenoid found in the endosperm of both the parental lines and their F1 hybrid progenies, constituting about 50% of their average total carotenoid content. Egesel et al. (2003) also found zeaxanthin as the major carotenoid, representing more than 50% of the total carotenoids, whereas Suwarno et al. (2014) reported 33% zeaxanthin in the F1 hybrids they evaluated. The contribution of β-carotene to the provitamin A content was larger than that of β-cryptoxanthin. This is in agreement with the results of Senete et al. (2011) but contrasts other studies that reported a larger proportion of β-cryptoxanthin relative to β-carotene (Egesel et al., 2003; Menkir et al., 2008; Suwarno et al., 2014).

The parental maize inbred lines used in our study contained both tropical and temperate germplasm in their genetic background. Both the pedigrees and the provitamin A content were used to plan the mating scheme that generated the F1 hybrids evaluated across the four environments (2 years × 2 locations). Our result did not reflect the heterotic patterns based on grain yield of the recurrent parents, as the donor lines might have disrupted the known pattern. Heterotic grouping of the provitamin A enriched inbred lines is therefore an ongoing work at IITA. In addition the provitamin A content of the hybrid progenies mirrored the profile of the parents irrespective of the genetic backgrounds. For instance, high provitamin A by high provitamin A crosses resulted in high provitamin A hybrids (set 1 hybrids), similarly low provitamin A by low provitamin A crosses resulted in low provitamin A content hybrid progenies (set of 5 hybrids), showing additive gene actions are controlling provitamin A content. More interestingly, a set of four hybrids with higher levels of provitamin A were generated by crossing low provitamin parents by high provitamin A parents, thus suggesting that breeders can use yellow maize inbred lines that have high breeding value for grain yield but have low provitamin A content as parents to develop viable provitamin A-biofortified hybrids for commercialization. The significant male and female GCA main effects for carotenoids in maize further confirms the importance of additive gene effects in controlling these traits. The significant SCA effect on lutein, β-cryptoxanthin, and provitamin A implies the presence of non-additive gene effects on the accumulation of these carotenoids. However, the GCA effects were larger than SCA effects for all carotenoids, suggesting the higher importance of additive gene action in the inheritance of carotenoid content in maize seed endosperm, which is consistent with results of other studies (Grogan et al., 1963; Egesel et al., 2003; Senete et al., 2011; Li et al., 2013; Suwarno et al., 2014). On the other hand, the predominance of SCA effects on carotenoid content has been demonstrated in other species, such as cabbage (Singh et al., 2011), cassava (Peninah et al., 2014), and cauliflower (Dey et al., 2014).

Our study identified inbred lines 1–8 as good combiners for heterosis effects on provitamin A carotenoids. The pattern of the GCA effects of the inbred parents generally followed the mean provitamin A concentrations for F1 hybrids within a set. Likewise, mean beta-carotene content in F1 hybrids paralleled the provitamin A content of their parental inbred lines. High and medium provitamin A parents were good combiners for provitamin A, whereas low provitamin A parents were poor combiners for provitamin A. Thus, crossing parents with good GCA and high provitamin A content can generate F1 hybrids with high provitamin A concentrations. However, our regression analyses suggested that provitamin A content in F1 hybrids may not always be predicted based on the provitamin A content of the parental lines, possibly because of the involvement of non-additive gene actions in regulating accumulations of provitamin A carotenoids. Therefore, both GCA and SCA should be considered when selecting inbred lines for high provitamin A F1 hybrid development (Dey et al., 2014).

The lack of correlation between carotenoids and important agronomic traits (including grain yield) indicates that carotenoid content in maize seed endosperm can be improved without adverse effects on agronomic performance of F1 hybrids, consistent with other studies (Egesel et al., 2003; Menkir et al., 2014; Suwarno et al., 2014). However, our findings are contradictory to the findings of Senete et al. (2011), who reported a significant negative correlation (*r* = −0.4) between grain yield and provitamin A content in maize. A higher SCA component of variance was observed for grain yield and other agronomic traits than for carotenoids, suggesting that non-additive gene action is important for the inheritance of agronomic traits.

While Burt et al. (2011) found heterosis for maize carotenoid content to be a rare phenomenon, Alfieri et al. (2014) found several hybrids manifesting both mid- and high parent heterosis. In our study, significant mid-parent heterosis was detected for provitamin A in 20 hybrids and for total carotenoids in 17 hybrids. However, very few hybrids displayed high parent heterosis for provitamin A and total carotenoids. Three hybrids (24, 32, and 52) were found to be superior in both agronomic performances and provitamin A content although they did not display significant mid- or high parent heterosis. We also identified two F1 hybrids (12 and 38) with significant high parent heterosis for provitamin A and superior agronomic performance.

In conclusion, our results demonstrate the feasibility of exploiting the heterotic potential of yellow and orange endosperm maize inbred lines for developing superior novel inbred lines and F1 hybrids with much higher concentrations of provitamin A and desirable agronomic characters to help combat vitamin A deficiency (particularly in poorer communities whose diets are predominantly maize dependent).

## Data Availability Statement

The raw data supporting the conclusions of this article will be made available by the authors, without undue reservation.

## Author Contributions

This work was conducted as part of GA's Ph.D. in the Genetics and Biotechnology Lab of CS at NUI Galway, in partnership with IITA. GA undertook the crossing activities, conducted the multi-location trials, carried out the statistical analysis, and wrote the first draft of the manuscript. AM contributed to the conception of the research, supplied the maize inbred lines used in the study, supervised the field work, and revised manuscript drafts. MG supervised and revised manuscript drafts. CS supervised GA Ph.D., provided project oversight and guidance, secured funding, revising, and finalization of the manuscript. AM and MG participated in the co-supervision with CS of GA Ph.D. All authors contributed to the article and approved the submitted version.

## Conflict of Interest

The authors declare that the research was conducted in the absence of any commercial or financial relationships that could be construed as a potential conflict of interest.

## Publisher's Note

All claims expressed in this article are solely those of the authors and do not necessarily represent those of their affiliated organizations, or those of the publisher, the editors and the reviewers. Any product that may be evaluated in this article, or claim that may be made by its manufacturer, is not guaranteed or endorsed by the publisher.
