# Supplementary material for: Combining Ability and Heterosis for Endosperm Carotenoids and Agronomic Traits in Tropical Maize Lines
Source: Front Plant Sci. 2021 Sep 9;12:674089. doi: 10.3389/fpls.2021.674089 (PMC8458911; doi:10.3389/fpls.2021.674089)
Supplement: Supplementary file 1 [file Data_Sheet_1.docx]

**Supplementary Data**

**Supplementary Table 1** Parental maize inbred lines used in a factorial mating scheme

| **Inbred line pedigree** | **Inbred line No.** | **Group^1^** | **Parentage^2^** | **Provitamin A ^3^** |
| --- | --- | --- | --- | --- |
| SYN-Y-STR-34-1-1-1-1-2-1-B*3/(DE3/CI7)/SYN-Y-STR-34-1-1-1-1-2-1-B*11 | 1 | I | F M | 10.14 |
| 4205/CI7/4205-6-B*6 | 2 | I | F M | 8.39 |
| KU1409/SC55/KU1409-4-B*4 | 3 | I | F M | 8.65 |
| SC55/KU1414-SR/KU1414-SR-6-B*6 | 4 | I | F M | 8.34 |
| (9450/KI 21-1-4-1-1-2-B/DE3/9450/KI 21-1-4-1-1-2-B) -29-B*3 | 5 | II | F M | 14.91 |
| 9450/KI21-3-2-2-1-3/KU1409/MO17LPA/KU1409-27-3-1-1-6-B*7 | 6 | II | F M | 15.72 |
| 9450/KI 21-1-5-3-2-2-B/DE3/9450/KI 21-1-5-3-2-2-B -32-B*4 | 7 | II | F M | 12.24 |
| 9450/KI 21-1-5-3-2-2-B/DE3/9450/KI 21-1-5-3-2-2-B -23-B*4 | 8 | II | F M | 11.31 |
| (9450/KI 21-1-4-1-1-1-B/DE3/9450/KI 21-1-4-1-1-1-B) -33-B*3 | 9 | III | F M | 8.59 |
| (9450 / KI 28)-1-2-1-1-B/DE3/(9450 / KI 28)-1-2-1-1-B -40-B*3 | 10 | III | F M | 12.72 |
| (9450/KI 21-1-4-1-1-2-B/DE3/9450/KI 21-1-4-1-1-2-B) -30-B*3 | 11 | III | F M | 10.80 |
| 9450/KI 21-1-4-1-1-1-B/DE3/9450/KI 21-1-4-1-1-1-B -26-B*4 | 12 | III | F M | 10.26 |
| (ACR97TZL-CCOMP1-Y-S3-13-1-B-B/CI7/ACR97TZL-CCOMP1-Y-S3-13-1-B-B)-13-B*3 | 13 | IV | M | 9.77 |
| (KU1409/SC55/KU1409)-S2-19-1-B*4 | 14 | IV | M | 7.64 |
| (POP66SR/ACR91SUWAN1-SRC1/ACR91SUWAN1-SRC1-6/(MP420/4001/MP420)-3-1-3-1-B)S2-5-B*6 | 15 | IV | M | 8.70 |
| (9071 / 4058)-8-2-1-1-B*6 | 16 | IV | M | 7.87 |
| (KU1414-SR/CI7/KU1414-SR) -49-B*3 | 17 | V | F M | 2.56 |
| (KU1414-SR/CI7/KU1414-SR) -35-B*3 | 18 | V | F M | 3.97 |
| (POP66SR/ACR91SUWAN1-SRC1/ACR91SUWAN1-SRC1-9 / (9450/CM 116/9450)-3-3-1-2-1)S2-5-B*4 | 19 | V | F M | 2.44 |
| (POP66SR/ACR91SUWAN1-SRC1/ACR91SUWAN1-SRC1-4 / 4001/KI21-4-1-1-1-1)S2-3-B*4 | 20 | V | F M | 3.35 |
| (9450/KI 21-1-4-1-1-1-B/DE3/9450/KI 21-1-4-1-1-1-B) -29-B*3 | 21 | VI | F | 1.99 |
| (9450 / KI 28)-1-2-1-1-B/DE3/(9450 / KI 28)-1-2-1-1-B -27-B*3 | 22 | VI | F | 2.17 |
| (9450 / KI 28)-1-2-1-1-B/DE3/(9450 / KI 28)-1-2-1-1-B -22-B*3 | 23 | VI | F | 2.91 |
| 9450/KI 21-1-5-3-2-1-B/DE3/9450/KI 21-1-5-3-2-1-B -27-B*4 | 24 | VI | F | 2.58 |
| 4001 | Check |  |  | -- |
| KU1414-SR | Check |  |  | -- |

^1^ Group number referring to groups each containing four sets of lines

^2^ Used either as F female (line) or M, male (tester) in different sets of crosses. Inbred lines 13 to 16 were used only as males, and inbred lines 21 to 24 were used only as females in the whole crossing scheme.

^3^ Provitamin A content data from preliminary carotenoid analyses.

**Supplementary Table 2** Combined ANOVA for carotenoid content of 24 yellow maize inbred lines (parents) and 4 checks evaluated across four environments

| **Variance Source** | **DF** | **Lutein** | **Zeaxanthin** | **β-cryptoxanthin** | **α-carotene** | **β-carotene** | **Provitamin A** | **Total carotenoid** |
| --- | --- | --- | --- | --- | --- | --- | --- | --- |
| **Envt** | 3 | 116.69** | 155.44** | 0.28** | 14.89** | 39.55** | 55.33** | 210.74** |
| **Rep(Envt)** | 4 | 7.35 | 7.96 | 0.02 | 0.5 | 0.4 | 0.56 | 32.72* |
| **Block (Envt*Rep)** | 24 | 4.23 | 10.08* | 0.01 | 0.96 | 0.35 | 0.97* | 25.25** |
| **Inbred line** | 27 | 87.36** | 117.32** | 0.09** | 5.87** | 9.35** | 10.75** | 165.47** |
| **Envt*Inbred line** | 80 | 5.19 | 7.29 | 0.02** | 0.9* | 2.04** | 1.9** | 16.27 |
| **Error** | 77 | 3.59 | 5.72 | 0.01 | 0.6 | 0.22 | 0.54 | 12.08 |
| **Corrected Total SS** | 215 | 3922.05 | 5331.5 | 7.05 | 394.31 | 625.74 | 749.18 | 8957.56 |
| **R^2^ %** |  | 0.93 | 0.92 | 0.88 | 0.88 | 0.97 | 0.94 | 0.90 |
| **CV%** |  | 33.89 | 22.70 | 38.51 | 36.10 | 18.92 | 20.12 | 16.61 |
| **Grand mean** |  | 5.59 | 10.53 | 2.02 | 0.29 | 2.49 | 3.65 | 20.92 |
| **H**±**SE** |  | 0.95±0.02 | 0.91±0.02 | 0.92±0.08 | 0.57±0.01 | 0.79±0.04 | 0.81±0.04 | 0.81±0.04 |

* Significant at P < 0.05; ** significant at *p<*0.01; Envt, Environment; Rep, Replication; DF, Degrees of Freedom; R^2^ %, percent explained variance; H, Heritability; SE, Standard Error

**Supplementary Table 3**. Carotenoid concentrations of parental inbred lines averaged across four environments

| **Line/ tester** | **Lutein** | **Zeaxanthin** | **α-carotene** | **β-crptoxanthin** | **β -carotene** | **Provitamin A** | **Total carotenoid** |
| --- | --- | --- | --- | --- | --- | --- | --- |
| 1 | 2.43±1.12 | 10.78±1.29 | 0.37±0.07 | 1.75±0.44 | 3.92±0.68 | 4.96±0.73 | 19.1±1.74 |
| 2 | 2.37±1.12 | 11.47±1.29 | 0.22±0.07 | 2.53±0.44 | 2.58±0.68 | 3.95±0.73 | 19.25±1.74 |
| 3 | 2.86±1.12 | 14.00±1.29 | 0.25±0.07 | 2.71±0.44 | 2.48±0.68 | 3.96±0.73 | 22.26±1.74 |
| 4 | 9.09±1.15 | 4.98±1.34 | 0.61±0.07 | 0.87±0.46 | 3.67±0.69 | 4.41±0.74 | 19.1±1.82 |
| 5 | 2.40±1.12 | 2.89±1.29 | 0.41±0.07 | 0.60±0.44 | 6.15±0.68 | 6.65±0.73 | 12.13±1.74 |
| 6 | 3.71±1.12 | 15.07±1.29 | 0.25±0.07 | 2.91±0.44 | 2.78±0.68 | 4.37±0.73 | 24.63±1.74 |
| 7 | 5.10±1.12 | 3.21±1.29 | 0.43±0.07 | 1.14±0.44 | 4.63±0.68 | 5.43±0.73 | 14.74±1.74 |
| 8 | 5.91±1.15 | 3.20±1.34 | 0.4±0.07 | 0.75±0.46 | 3.35±0.69 | 3.92±0.74 | 13.71±1.82 |
| 9 | 6.04±1.12 | 9.14±1.29 | 0.32±0.07 | 3.59±0.44 | 1.80±0.68 | 3.73±0.73 | 20.93±1.74 |
| 10 | 4.44±1.22 | 11.54±1.41 | 0.3±0.08 | 2.71±0.48 | 2.44±0.75 | 3.96±0.79 | 21.63±1.94 |
| 11 | 9.14±1.15 | 13.07±1.34 | 0.44±0.07 | 2.46±0.46 | 2.40±0.69 | 3.86±0.74 | 27.52±1.82 |
| 12 | 4.37±1.12 | 15.08±1.29 | 0.33±0.07 | 4.80±0.44 | 3.21±0.68 | 5.78±0.73 | 27.62±1.74 |
| 13 | 7.10±1.12 | 16.31±1.29 | 0.38±0.07 | 2.47±0.44 | 3.39±0.68 | 4.79±0.73 | 29.50±1.74 |
| 14 | 4.14±1.12 | 17.4±1.29 | 0.22±0.07 | 1.97±0.44 | 3.19±0.68 | 4.31±0.73 | 27.10±1.74 |
| 15 | 4.50±1.12 | 12.2±1.29 | 0.4±0.07 | 3.03±0.44 | 2.67±0.68 | 4.39±0.73 | 22.88±1.74 |
| 16 | 4.89±1.12 | 15.1±1.29 | 0.21±0.07 | 1.96±0.44 | 1.71±0.68 | 2.80±0.73 | 23.91±1.74 |
| 17 | 2.79±1.15 | 9.65±1.34 | 0.11±0.07 | 1.05±0.46 | 1.10±0.69 | 1.73±0.74 | 14.74±1.82 |
| 18 | 4.67±1.12 | 11.22±1.29 | 0.15±0.07 | 1.36±0.44 | 1.22±0.68 | 1.97±0.73 | 18.7±1.74 |
| 19 | 5.71±1.12 | 8.18±1.29 | 0.2±0.07 | 1.05±0.44 | 1.24±0.68 | 1.88±0.73 | 16.34±1.74 |
| 20 | 4.69±1.12 | 9.32±1.29 | 0.33±0.07 | 2.40±0.44 | 2.81±0.68 | 4.18±0.73 | 19.63±1.74 |
| 21 | 13.06±1.12 | 7.07±1.29 | 0.26±0.07 | 0.94±0.44 | 1.19±0.68 | 1.78±0.73 | 22.49±1.74 |
| 22 | 3.13±1.12 | 6.25±1.29 | 0.22±0.07 | 2.08±0.44 | 2.07±0.68 | 3.23±0.73 | 13.82±1.74 |
| 23 | 19.19±1.12 | 6.97±1.29 | 0.43±0.07 | 1.04±0.44 | 2.18±0.68 | 2.9±0.73 | 29.74±1.74 |
| 24 | 3.24±1.19 | 5.50±1.39 | 0.13±0.07 | 1.55±0.47 | 0.81±0.7 | 1.68±0.75 | 11.56±1.91 |
| Check1 | 5.77±1.12 | 12.77±1.29 | 0.235±0.07 | 2.485±0.44 | 1.71±0.68 | 3.075±0.73 | 22.915±1.74 |
| Check2 | 4.21±1.12 | 12.9±1.29 | 0.13±0.07 | 1.855±0.44 | 1.725±0.68 | 2.72±0.73 | 20.915±1.74 |
| Grand Mean | 5.53±1.13 | 10.39±1.31 | 0.29±0.07 | 2.01±0.45 | 2.49±0.69 | 3.65±0.73 | 20.74±1.77 |
| SD | 3.56 | 4.16 | 0.12 | 0.96 | 1.18 | 1.27 | 5.10 |
| Range | 2.37-19.19 | 2.89-17.4 | 0.11-0.61 | 0.6-4.8 | 0.81-6.15 | 1.68-6.65 | 11.56-29.74 |
| LSD(p<0.05)^2^ | 2.38 | 2.82 | 0.16 | 0.98 | 1.52 | 1.46 | 1.60 |

^1^ Haplotypes of the parents for the favorable/unfavorable alleles of the most significantly associated polymorphisms, 3’TE and 5’TE, of the gene *crtRB1* as verified in Azmach et al., 2013. Allele 3 and 1 for 3’TE represent unfavorable and favorable alleles, respectively 2 and 1 represented favorable and unfavorable alleles for the 5’TE polymorphism.

^2^ average LSD at p<0.05

**Supplementary Table 4**. Carotenoid concentration (µg/g) of 80 hybrids generated from 24 inbred lines in a factorial mating design and tested across environments at Saminaka and Zaria in 2012 and 2013.

| Hybrid | Lutein | Zeaxanthin | α-carotene | β-crptoxanthin | β-carotene | Provitamin A | Total carotenoid |
| --- | --- | --- | --- | --- | --- | --- | --- |
| 1 | 4.18 | 6.3 | 0.34 | 1.43 | 4.06 | 4.93 | 15.85 |
| 2 | 5.27 | 3.88 | 0.31 | 1.48 | 3.32 | 4.21 | 13.66 |
| 3 | 6.65 | 8.01 | 0.5 | 2.28 | 4.42 | 5.82 | 21.07 |
| 4 | 7.86 | 4.99 | 0.38 | 0.93 | 4.17 | 4.83 | 18.2 |
| 5 | 5.08 | 5.94 | 0.37 | 1.14 | 3.9 | 4.64 | 15.96 |
| 6 | 4.31 | 5.65 | 0.35 | 1.24 | 3.71 | 4.51 | 15 |
| 7 | 5.41 | 8.62 | 0.38 | 1.59 | 3.82 | 4.81 | 19.37 |
| 8 | 8.92 | 4.91 | 0.44 | 0.71 | 4.72 | 5.29 | 19.49 |
| 9 | 5.35 | 12.72 | 0.32 | 2.33 | 3.71 | 5.03 | 23.56 |
| 10 | 3.6 | 14.31 | 0.27 | 2.52 | 3.11 | 4.49 | 22.99 |
| 11 | 4.5 | 17.04 | 0.34 | 2.89 | 2.91 | 4.53 | 26.89 |
| 12 | 7.17 | 12.67 | 0.46 | 1.96 | 4.84 | 6.06 | 26.58 |
| 13 | 5.17 | 8.33 | 0.41 | 1.48 | 4.9 | 5.85 | 19.94 |
| 14 | 3.57 | 7.57 | 0.3 | 1.62 | 4.93 | 5.89 | 17.47 |
| 15 | 3.76 | 11.57 | 0.33 | 2.15 | 5.04 | 6.29 | 22.04 |
| 16 | 6.28 | 5.51 | 0.44 | 0.84 | 5.33 | 5.97 | 18.08 |
| 17 | 8.08 | 8.78 | 0.4 | 1.78 | 2.87 | 3.95 | 21.46 |
| 18 | 7.89 | 10.53 | 0.44 | 1.65 | 3.91 | 4.95 | 23.91 |
| 19 | 9.86 | 10.84 | 0.45 | 1.58 | 3.73 | 4.76 | 25.89 |
| 20 | 8.71 | 13.56 | 0.42 | 2.15 | 4.15 | 5.43 | 28.37 |
| 21 | 6.51 | 12.2 | 0.33 | 3.11 | 2.69 | 4.41 | 23.69 |
| 22 | 3.92 | 14.84 | 0.36 | 3.52 | 3.32 | 5.27 | 25.03 |
| 23 | 7.21 | 14.53 | 0.53 | 3.76 | 3.86 | 6 | 28.6 |
| 24 | 4.14 | 13.71 | 0.3 | 3.81 | 3.24 | 5.29 | 24.08 |
| 25 | 5.24 | 9.12 | 0.26 | 2.23 | 2.6 | 3.84 | 18.59 |
| 26 | 3.91 | 11.68 | 0.31 | 2.69 | 2.7 | 4.19 | 20.55 |
| 27 | 5.16 | 11.34 | 0.37 | 3.08 | 3.01 | 4.72 | 21.9 |
| 28 | 3.59 | 11.12 | 0.22 | 3.28 | 2.88 | 4.62 | 20.12 |
| 29 | 5.1 | 9.17 | 0.36 | 2.45 | 2.71 | 4.11 | 19.07 |
| 30 | 5.1 | 14.04 | 0.42 | 2.63 | 3.86 | 5.39 | 25.24 |
| 31 | 6.3 | 13.32 | 0.42 | 2.93 | 3.89 | 5.57 | 25.77 |
| 32 | 4.14 | 14.19 | 0.33 | 3.42 | 3.58 | 5.44 | 24.57 |
| 33 | 5.02 | 12.55 | 0.28 | 2.36 | 3.74 | 5.04 | 23.11 |
| 34 | 5.14 | 14.84 | 0.29 | 2.54 | 2.4 | 3.82 | 24.43 |
| 35 | 5.41 | 9.82 | 0.3 | 1.69 | 2.92 | 3.92 | 19.66 |
| 36 | 4.19 | 8.49 | 0.32 | 2.05 | 3.28 | 4.48 | 17.69 |
| 37 | 4.17 | 10.99 | 0.36 | 3.16 | 4.74 | 6.53 | 22.68 |
| 38 | 4.8 | 14.54 | 0.35 | 3.6 | 3.36 | 5.32 | 25.44 |
| 39 | 4.29 | 7.65 | 0.32 | 1.92 | 2.99 | 4.11 | 16.44 |
| 40 | 6.24 | 7.34 | 0.45 | 2.38 | 3.66 | 5.07 | 19.18 |
| 41 | 3.61 | 13.48 | 0.26 | 1.51 | 4.19 | 5.08 | 22.48 |
| 42 | 3.88 | 17.17 | 0.3 | 2.36 | 3 | 4.34 | 26.06 |
| 43 | 4.76 | 9.97 | 0.23 | 1.35 | 3.24 | 4.03 | 19.16 |
| 44 | 7.3 | 7.74 | 0.33 | 1.5 | 3.12 | 4.03 | 19.47 |
| 45 | 5.79 | 11.1 | 0.36 | 2.19 | 4.16 | 5.44 | 22.91 |
| 46 | 4.39 | 12.93 | 0.28 | 2.68 | 3.15 | 4.63 | 22.65 |
| 47 | 5.42 | 7.95 | 0.33 | 1.64 | 3.65 | 4.64 | 18.31 |
| 48 | 6.79 | 7.2 | 0.37 | 1.89 | 3.06 | 4.19 | 18.9 |
| 49 | 4.05 | 12.76 | 0.19 | 2.46 | 2.32 | 3.65 | 20.9 |
| 50 | 5.01 | 13.66 | 0.23 | 3.28 | 2.45 | 4.19 | 23.5 |
| 51 | 4.71 | 10.8 | 0.25 | 2.93 | 2.29 | 3.88 | 20.11 |
| 52 | 4.66 | 11.08 | 0.3 | 3.46 | 3.36 | 5.25 | 22 |
| 53 | 6.05 | 11.3 | 0.36 | 2.13 | 2.26 | 3.51 | 21.41 |
| 54 | 9.59 | 14.38 | 0.41 | 2.37 | 2.35 | 3.74 | 28.36 |
| 55 | 6.84 | 10.88 | 0.31 | 2.21 | 2.29 | 3.56 | 21.94 |
| 56 | 8.18 | 12.38 | 0.47 | 2.96 | 3.26 | 4.98 | 26.27 |
| 57 | 3.66 | 14.21 | 0.22 | 3.44 | 2.4 | 4.22 | 22.63 |
| 58 | 4.71 | 14.75 | 0.27 | 2.59 | 2.53 | 3.97 | 24.08 |
| 59 | 5.21 | 10.52 | 0.29 | 2.38 | 2.38 | 3.73 | 19.99 |
| 60 | 5.22 | 8.84 | 0.29 | 2.82 | 2.86 | 4.41 | 19.21 |
| 61 | 5.88 | 10.24 | 0.25 | 2.06 | 1.93 | 3.08 | 19.79 |
| 62 | 5.63 | 11.05 | 0.27 | 2.27 | 1.98 | 3.27 | 20.61 |
| 63 | 5.65 | 8.05 | 0.32 | 2.08 | 1.69 | 2.9 | 17.21 |
| 64 | 5.79 | 7.92 | 0.23 | 2.56 | 2.1 | 3.48 | 17.64 |
| 65 | 9.37 | 7.14 | 0.35 | 1.18 | 2.21 | 2.99 | 19.78 |
| 66 | 4.25 | 8.25 | 0.28 | 1.95 | 2.51 | 3.64 | 16.71 |
| 67 | 8.87 | 7.9 | 0.33 | 1.44 | 2.9 | 3.79 | 21.02 |
| 68 | 5.48 | 10.81 | 0.21 | 2.15 | 2.6 | 3.78 | 20.71 |
| 69 | 9.8 | 5.53 | 0.24 | 0.64 | 0.99 | 1.44 | 17.07 |
| 70 | 5.92 | 9.06 | 0.22 | 1.64 | 2.13 | 3.06 | 18.28 |
| 71 | 9.09 | 6.45 | 0.32 | 1.24 | 1.74 | 2.52 | 18.53 |
| 72 | 4.26 | 9.91 | 0.12 | 1.49 | 1.19 | 1.98 | 16.39 |
| 73 | 9.54 | 7.25 | 0.3 | 0.97 | 1.4 | 2.04 | 19.25 |
| 74 | 4.46 | 10.14 | 0.29 | 2.02 | 2.36 | 3.5 | 18.75 |
| 75 | 9.68 | 9.94 | 0.41 | 1.43 | 1.98 | 2.9 | 23.16 |
| 76 | 4.01 | 12.37 | 0.18 | 1.74 | 1.49 | 2.45 | 19.24 |
| 77 | 8.65 | 7.9 | 0.29 | 0.98 | 1.64 | 2.26 | 18.96 |
| 78 | 3.5 | 10.69 | 0.25 | 1.83 | 2.41 | 3.46 | 18.38 |
| 79 | 9.11 | 10.09 | 0.38 | 1.55 | 2.28 | 3.25 | 23.04 |
| 80 | 4.5 | 12.99 | 0.17 | 1.61 | 1.35 | 2.24 | 20.19 |
| 81 (Check) | 4.47 | 14.4 | 0.18 | 2.01 | 1.73 | 2.83 | 22.33 |
| Grand Mean | 5.79 | 10.47 | 0.32 | 2.14 | 3.04 | 4.27 | 21.1 |
| Average SE | 1.02 | 1.23 | 0.07 | 0.3 | 0.64 | 0.72 | 2.06 |
| Min | 3.5 | 3.88 | 0.12 | 0.64 | 0.99 | 1.44 | 13.66 |
| Max | 9.86 | 17.17 | 0.53 | 3.81 | 5.33 | 6.53 | 28.6 |
| LSD  (p<0.01) | 1.88 | 2.6 | 0.15 | 1.25 | 0.6 | 1.34 | 4.12 |

**Supplementary Table 5** Estimates of SCA effects for carotenoid contents in 80 maize hybrids generated through factorial mating of 24 inbred lines

| **Hybrid** | **Set** | **Lutein** | **Zeaxanthin** | **β-cryptoxanthin** | **β-carotene** | **Provitamin A** | **Total carotenoid** |
| --- | --- | --- | --- | --- | --- | --- | --- |
| 1 | 1 | -0.91 | 2.78** | 0.48** | -1** | -1** | 1.21 |
| 2 | 1 | 0.99 | 0.74 | 0.36* | -1.38** | -1.38** | 0.52 |
| 3 | 1 | 1.46* | 1.3 | 0.68** | -0.53* | -0.53* | 2.86* |
| 4 | 1 | 0.02 | 2.59** | 0.42** | -1.54** | -1.54** | 1.31 |
| 5 | 1 | 0 | 1.76 | 0.51** | -1.25** | -1.25** | 0.8 |
| 6 | 1 | 0.02 | 2.12* | 0.52** | -1.04** | -1.04** | 1.55 |
| 7 | 1 | 0.28 | 1.45 | 0.34* | -1.18** | -1.18** | 0.8 |
| 8 | 1 | 1.26* | 2.07* | 0.57** | -0.98** | -0.98** | 2.76 |
| 9 | 1 | 1.14 | 0.77 | 0.43** | -1.01** | -1.01** | 1.17 |
| 10 | 1 | 0.02 | 2.56** | 0.52** | -1.21** | -1.21** | 1.8 |
| 11 | 1 | 0.02 | 2.1* | 0.42** | -1.74** | -1.74** | 0.64 |
| 12 | 1 | 0.37 | 1.97* | 0.57** | -0.5* | -0.5 | 2.3 |
| 13 | 1 | 1.33* | 2.09* | 0.52** | -1.2** | -1.2** | 2.73 |
| 14 | 1 | 0.52 | 1.99* | 0.54** | -0.82** | -0.82** | 2.04 |
| 15 | 1 | -0.2 | 2.55** | 0.5** | -1** | -1** | 1.61 |
| 16 | 1 | -0.09 | 0.78 | 0.38* | -1.44** | -1.44** | -0.46 |
| 17 | 3 | 1.25* | -0.59 | 0.05 | -0.43* | -0.43 | 0.16 |
| 18 | 3 | 1.49* | -0.9 | -0.27 | -0.58** | -0.58* | -0.11 |
| 19 | 3 | 1.34* | 0.03 | 0.04 | -0.32 | -0.32 | 1.12 |
| 20 | 3 | -1.1 | -0.3 | 0.05 | -0.06 | -0.06 | -1.41 |
| 21 | 3 | 0.58 | -0.62 | 0.26 | -0.12 | -0.12 | 0.13 |
| 22 | 3 | 1.1 | -0.21 | 0.17 | -0.2 | -0.2 | 0.76 |
| 23 | 3 | 0.24 | -0.98 | -0.36* | -0.83** | -0.83** | -1.77 |
| 24 | 3 | 1.06 | 0.04 | -0.21 | -0.24 | -0.24 | 0.63 |
| 25 | 3 | -0.13 | -0.32 | -0.35* | -0.35 | -0.35 | -1.11 |
| 26 | 3 | 0.22 | 0.62 | 0 | -0.35 | -0.35 | 0.53 |
| 27 | 3 | 0.72 | -0.57 | 0.17 | -0.25 | -0.25 | 0.03 |
| 28 | 3 | 2.17** | -1.48 | 0.04 | -0.45* | -0.45 | 0.3 |
| 29 | 3 | 1.28* | -0.22 | -0.09 | -0.5* | -0.5 | 0.58 |
| 30 | 3 | 0.17 | -1.27 | -0.04 | -0.26 | -0.26 | -1.43 |
| 31 | 3 | 0.68 | -0.24 | 0.01 | 0.01 | 0.01 | 0.37 |
| 32 | 3 | 0.86 | -0.02 | -0.02 | -0.64** | -0.64* | 0.23 |
| 33 | 2 | -0.98 | -1.48 | -0.22 | -0.42* | -0.42 | -2.94* |
| 34 | 2 | -0.15 | -2.67** | -0.64** | -0.16 | -0.16 | -3.5* |
| 35 | 2 | -0.12 | -2.21* | -0.95** | -0.55** | -0.55* | -3.68* |
| 36 | 2 | 0.69 | -0.09 | -0.67** | 0.06 | 0.06 | 0.25 |
| 37 | 2 | 0.62 | -0.99 | -0.7** | -0.24 | -0.24 | -1.18 |
| 38 | 2 | -0.96 | -1.36 | -0.5** | -0.37 | -0.37 | -3.08* |
| 39 | 2 | 0.37 | -1.3 | -0.5** | 0 | 0 | -1.18 |
| 40 | 2 | -0.59 | -2.79** | -0.77** | -0.45* | -0.45 | -4.43** |
| 41 | 2 | 0.26 | -1.07 | -0.9** | 0.04 | 0.04 | -1.57 |
| 42 | 2 | 0.06 | -1.34 | -0.61** | -0.45* | -0.45 | -2.05 |
| 43 | 2 | -0.64 | -1.73 | -0.43** | -0.35 | -0.35 | -3* |
| 44 | 2 | -0.24 | -2.3* | -0.54** | -0.3 | -0.3 | -3.25* |
| 45 | 2 | -0.47 | -2.91** | -0.66** | -0.45* | -0.45 | -4.19** |
| 46 | 2 | 0.49 | -1.07 | -0.72** | -0.08 | -0.08 | -1.25 |
| 47 | 2 | -0.17 | -1.2 | -0.6** | -0.16 | -0.16 | -2 |
| 48 | 2 | -0.41 | -1.27 | -0.5** | -0.37 | -0.37 | -2.43 |
| 49 | 4 | 0.29 | -1.03 | -0.98** | 0.5* | 0.5 | -0.97 |
| 50 | 4 | -0.01 | -1.38 | -0.23 | 0.6** | 0.6* | -0.99 |
| 51 | 4 | 0.33 | -0.98 | -0.37* | 0.58** | 0.58* | -0.25 |
| 52 | 4 | -0.15 | -0.71 | -0.36* | 0.89** | 0.89** | -0.16 |
| 53 | 4 | -0.75 | -2.51** | -0.65** | 0.55** | 0.55* | -2.92* |
| 54 | 4 | 1.51* | -1.05 | -0.58** | 0.51* | 0.51* | 0.53 |
| 55 | 4 | -0.67 | -1.07 | -0.46** | 0.65** | 0.65* | -1.54 |
| 56 | 4 | 0.37 | 0.52 | -0.26 | 0.86** | 0.86** | 1.55 |
| 57 | 4 | -0.17 | 0.25 | 0.24 | 0.65** | 0.65* | 0.63 |
| 58 | 4 | -0.46 | -0.36 | -0.7** | 0.7** | 0.7** | -0.52 |
| 59 | 4 | 0.78 | -1.2 | -0.66** | 0.75** | 0.75** | -0.11 |
| 60 | 4 | 0.32 | -2.81** | -0.83** | 0.47* | 0.47 | -2.38 |
| 61 | 4 | 1.09 | -0.82 | -0.56** | 0.87** | 0.87** | 0.88 |
| 62 | 4 | -0.58 | -1.32 | -0.43** | 0.76** | 0.76** | -1.4 |
| 63 | 4 | 0.03 | -0.85 | -0.46** | 0.59** | 0.59* | -0.48 |
| 64 | 4 | -0.08 | -1.11 | -0.5** | 0.34 | 0.34 | -1.38 |
| 65 | 5 | -1.16 | 2.04* | 0.7** | 1.12** | 1.12** | 2.54 |
| 66 | 5 | -1.42* | 0.47 | 0.58** | 0.62** | 0.62* | -0.02 |
| 67 | 5 | -1.5* | 1.37 | 0.49** | 1.17** | 1.17** | 1.37 |
| 68 | 5 | -0.38** | 1.02 | 0.86** | 1.42** | 1.42** | 2.68 |
| 69 | 5 | -1.03 | 1.28 | 0.6** | 0.94** | 0.94** | 1.63 |
| 70 | 5 | -0.12 | 2.16* | 0.67** | 1.33** | 1.33** | 3.84** |
| 71 | 5 | -1.56** | 0.46 | 0.71** | 1.02** | 1.02** | 0.39 |
| 72 | 5 | -1.73** | 1.01 | 0.64** | 1.05** | 1.05** | 0.72 |
| 73 | 5 | -0.91 | 0.74 | 0.64** | 1.1** | 1.1** | 1.39 |
| 74 | 5 | -1.29* | 1.01 | 0.73** | 1.19** | 1.19** | 1.45 |
| 75 | 5 | -0.65 | 1.69 | 0.63** | 1** | 1** | 2.42 |
| 76 | 5 | -1.59** | 1.46 | 0.61** | 1.04** | 1.04** | 1.32 |
| 77 | 5 | -1.34* | 0.84 | 0.68** | 1.17** | 1.17** | 1.02 |
| 78 | 5 | -1.62** | 1.26 | 0.64** | 1.19** | 1.19** | 1.31 |
| 79 | 5 | -0.74 | 1.39 | 0.79** | 1.15** | 1.15** | 2.4 |
| 80 | 5 | -0.75 | 1.42 | 0.51** | 0.82** | 0.82** | 1.86 |
|  |  | 0.59 | 0.91 | 0.15 | 0.21 | 0.26 | 1.43 |

### * significant at P < 0.05, ** significant at *p<*0.01 SE = Standard error, GM = Grand mean

**Supplementary Table 6** ANOVA for agronomic traits of the 24 parental inbred lines and 4 checks evaluated across environments

| **Variance Source** | **AD** | **SD** | **ASI** | **EH** | **PH** | **HC** | **PA** | **EA** |
| --- | --- | --- | --- | --- | --- | --- | --- | --- |
| **Envt** | 246.13** | 268.97** | 18.48** | 2488.65** | 10497.57** | 20.37** | 4.48** | 9.63** |
| **Rep(Envt)** | 14.28** | 24.21** | 2.54 | 308.86* | 307.01 | 0.96** | 0.25 | 0.1 |
| **Block(Envt*Rep)** | 5.33** | 7.12* | 2.05* | 156.42* | 398.78** | 0.15 | 0.21 | 0.3 |
| **Inbred line** | 58.13** | 86.01** | 6.63** | 371.41** | 880.55** | 0.53** | 0.93** | 0.63** |
| **Envt*Inbred line** | 4.71** | 7.3** | 2.24** | 72.21 | 174.43 | 0.33** | 0.37** | 0.28 |
| **Error** | 210.04 | 310.23 | 1.14 | 93.55 | 127.13 | 0.17 | 0.19 | 0.29 |
| **Corrected Total SS** | 3555.55 | 4769.83 | 562.92 | 39265.28 | 104487.73 | 133.84 | 101.25 | 80.99 |
| **R^2^ %** | 0.94 |  | 0.84 | 0.80 | 0.90 | 0.89 | 0.84 | 0.78 |
| **CV %** | 2.43 |  | 42.07 | 16.20 | 8.42 | 22.61 | 13.66 | 15.33 |
| **Grand mean** | 64.96 |  | 2.54 | 59.72 | 133.88 | 1.85 | 3.19 | 3.49 |
| **H**±**SE** | 0.92±0.02 | 0.92±0.02 | 0.69±0.1 | 0.83±0.05 | 0.79±0.06 | 0.37±0.2 | 0.64±0.12 | 0.54±0.19 |

* significant at P < 0.05, ** significant at *p<*0.01; AD, no. of days to anthesis; SD, no. of days to silking; ASI, anthesis silking interval; EH, Ear height; PH, plant height; PA, plant aspect and EA, ear aspect; Envt, Environment; Rep, Replication; DF, Degrees of Freedom; R2 %, percent explained variance; H, Heritability; SE, Standard Error

**Supplementary Table 7.** Mean agronomic performances of 81 maize hybrids

| Hybrid | AD | PH | EH | GY | EA | PA |
| --- | --- | --- | --- | --- | --- | --- |
| 1 | 58.25 | 189.03 | 97.88 | 4.68 | 2.95 | 3.18 |
| 2 | 57.56 | 201.45 | 110.68 | 4.77 | 3.02 | 3.26 |
| 3 | 60.01 | 191.87 | 105.64 | 4.73 | 3.2 | 3.37 |
| 4 | 59.82 | 187.61 | 107.86 | 4.29 | 3.37 | 3.12 |
| 5 | 57.69 | 184.75 | 79.79 | 4.41 | 3.25 | 3.23 |
| 6 | 56.41 | 198.75 | 90.83 | 5.08 | 2.72 | 3.05 |
| 7 | 56.02 | 195.35 | 88.6 | 5.31 | 2.61 | 2.69 |
| 8 | 57.15 | 194.11 | 84.98 | 4.59 | 3.3 | 2.48 |
| 9 | 59.68 | 183.61 | 84.47 | 2.62 | 3.81 | 3.78 |
| 10 | 59.68 | 182.28 | 90.06 | 2.76 | 3.72 | 3.94 |
| 11 | 62.43 | 147.7 | 68.4 | 0.66 | 4.36 | 4.34 |
| 12 | 57.87 | 221.66 | 105.23 | 4.89 | 2.59 | 2.58 |
| 13 | 56.32 | 194.39 | 87.69 | 4.98 | 3.3 | 3.62 |
| 14 | 56.64 | 197.55 | 96.73 | 5.29 | 3.08 | 3.09 |
| 15 | 56.98 | 200.1 | 86.57 | 4.66 | 3.08 | 2.89 |
| 16 | 57.88 | 194.17 | 89.28 | 4.56 | 3.4 | 2.91 |
| 17 | 58.52 | 194.23 | 90.42 | 3.83 | 3.22 | 2.83 |
| 18 | 60.96 | 201.34 | 106.98 | 4.23 | 3.21 | 3.1 |
| 19 | 58.02 | 183.55 | 83.92 | 4.06 | 2.77 | 3.17 |
| 20 | 58.47 | 196.97 | 90.75 | 4.28 | 3.12 | 2.83 |
| 21 | 58 | 194.67 | 86.51 | 4.16 | 2.83 | 3.42 |
| 22 | 60.21 | 215.02 | 100.3 | 4.43 | 3.1 | 3.34 |
| 23 | 56.93 | 194.1 | 85.64 | 4.65 | 2.77 | 2.96 |
| 24 | 56.47 | 209.85 | 87.17 | 5.71 | 2.64 | 2.71 |
| 25 | 58.15 | 201.78 | 92.13 | 4.92 | 2.82 | 3.15 |
| 26 | 59.34 | 209.83 | 105.35 | 4.29 | 3.18 | 3.46 |
| 27 | 57.2 | 188.9 | 84.63 | 4.9 | 2.83 | 3.3 |
| 28 | 57.04 | 206.02 | 97.16 | 5.42 | 2.95 | 2.75 |
| 29 | 57.62 | 187.4 | 80.83 | 4.53 | 3.03 | 3.31 |
| 30 | 59.92 | 201.77 | 95.08 | 3.73 | 3.4 | 3.42 |
| 31 | 56.55 | 186.59 | 79.77 | 4.74 | 2.88 | 3.18 |
| 32 | 56.84 | 199.04 | 86.82 | 5.87 | 2.47 | 2.99 |
| 33 | 58.36 | 178.27 | 83.27 | 5.23 | 3.03 | 2.68 |
| 34 | 58.77 | 209.27 | 98.31 | 4.98 | 2.67 | 2.88 |
| 35 | 57.48 | 175.69 | 79.61 | 4.39 | 3.3 | 2.81 |
| 36 | 60.62 | 180.62 | 95.91 | 4.26 | 3.31 | 3.29 |
| 37 | 56.26 | 175.94 | 83.13 | 4.21 | 3.44 | 3.29 |
| 38 | 57.45 | 196.81 | 93.67 | 5.23 | 2.3 | 2.45 |
| 39 | 56.02 | 173.76 | 79.99 | 4.3 | 3.03 | 2.49 |
| 40 | 59.11 | 185.89 | 100.67 | 3.8 | 3.49 | 3.12 |
| 41 | 56.98 | 181.78 | 81.76 | 4.14 | 3.46 | 3.28 |
| 42 | 57.44 | 182.3 | 86.03 | 4.97 | 2.82 | 3.19 |
| 43 | 56.62 | 180.84 | 81.85 | 4.63 | 2.83 | 2.9 |
| 44 | 59.05 | 168.04 | 94.19 | 4.4 | 3.19 | 3.3 |
| 45 | 58.61 | 198.5 | 94.19 | 4.88 | 3.23 | 3.21 |
| 46 | 57.48 | 220.75 | 109.98 | 5.35 | 2.42 | 3.09 |
| 47 | 57.24 | 206.28 | 90.18 | 4.93 | 3 | 3.23 |
| 48 | 60.19 | 206.32 | 107.91 | 4.31 | 3.61 | 3.39 |
| 49 | 58.34 | 192.81 | 89.3 | 4.57 | 2.83 | 2.65 |
| 50 | 59.34 | 186.6 | 84.5 | 4.41 | 3.07 | 2.67 |
| 51 | 57.55 | 189.51 | 85.17 | 4.91 | 2.98 | 3.35 |
| 52 | 57.37 | 196.48 | 88.01 | 5.68 | 2.34 | 2.73 |
| 53 | 58.06 | 190.95 | 87.05 | 4.59 | 2.89 | 2.99 |
| 54 | 58.99 | 183.59 | 85.84 | 4.69 | 2.99 | 2.66 |
| 55 | 56.34 | 174.79 | 72.73 | 4.93 | 3.13 | 3.13 |
| 56 | 57.97 | 183.97 | 79.8 | 4.79 | 2.89 | 3.06 |
| 57 | 60.35 | 200.25 | 98.74 | 3.93 | 3.43 | 3.29 |
| 58 | 61.03 | 209.02 | 105.3 | 4.54 | 3.16 | 3.07 |
| 59 | 58.67 | 195.59 | 100.3 | 5.34 | 2.93 | 2.87 |
| 60 | 59.18 | 204.6 | 98.36 | 4.64 | 2.96 | 3.43 |
| 61 | 60.24 | 191.49 | 89.77 | 3.27 | 3.29 | 3.09 |
| 62 | 59.12 | 188.14 | 88.24 | 4.76 | 2.91 | 2.99 |
| 63 | 59.15 | 186.42 | 82.18 | 3.77 | 3.34 | 3.23 |
| 64 | 58.62 | 189.02 | 85.66 | 5.22 | 2.75 | 2.99 |
| 65 | 56.29 | 189.72 | 87.12 | 5.11 | 2.87 | 3.16 |
| 66 | 58.07 | 189.92 | 90.69 | 5.05 | 2.99 | 3.21 |
| 67 | 59.32 | 202.59 | 100.79 | 4.79 | 3.01 | 3.33 |
| 68 | 61.8 | 196 | 96.33 | 4.6 | 3.16 | 3.46 |
| 69 | 56.39 | 179.09 | 81.24 | 5.67 | 2.71 | 2.78 |
| 70 | 58.59 | 185.9 | 100.5 | 4.84 | 3 | 3.13 |
| 71 | 60.22 | 190.87 | 93.3 | 5.03 | 3.06 | 3.44 |
| 72 | 61.49 | 181.17 | 84.24 | 4.1 | 3.03 | 3.24 |
| 73 | 57.86 | 198.7 | 95.55 | 5.17 | 2.66 | 2.55 |
| 74 | 59.57 | 200.69 | 111.47 | 4.7 | 2.72 | 2.82 |
| 75 | 60.77 | 205.1 | 112.59 | 4.64 | 2.96 | 3.24 |
| 76 | 63.34 | 191.75 | 87.64 | 3.52 | 3.11 | 2.7 |
| 77 | 57.22 | 192.72 | 87.68 | 4.66 | 2.64 | 2.95 |
| 78 | 59.49 | 206.43 | 112.6 | 4.52 | 3.04 | 3.26 |
| 79 | 60.68 | 210.85 | 108.89 | 4.55 | 2.89 | 3.06 |
| 80 | 63.19 | 188.97 | 91.8 | 3.49 | 3.34 | 3.03 |
| 81 | 58.99 | 182.77 | 96.18 | 4.86 | 2.45 | 2.98 |
| Grand Mean | 58.54 | 192.75 | 91.95 | 4.56 | 3.03 | 3.09 |
| Min | 56.02 | 147.7 | 68.4 | 0.66 | 2.3 | 2.45 |
| Max | 63.34 | 221.66 | 112.6 | 5.87 | 4.36 | 4.34 |
| SE | 1.63 | 10 | 7.02 | 1.25 | 0.22 | 0.2 |
| LSD (*P*<0.01) | 1.64 | 16.8 | 11.27 | 1.26 | 0.66 | 0.67 |

SD, no. of days to silking; AD, no. of days to anthesis; PH, plant height in cm; EH, ear height in cm; GY, grain yield in t/ha; EA, ear aspect (1 to 5 scale score); PA, plant aspect (1 to 5 scale score); Min, Minimum; Max, Maximum;

**Supplementary Table 8.** SCA effects for agronomic traits in 80 maize hybrid progenies generated through factorial mating of 24 inbred lines

| **Hybrid** | **Set** | **SD** | **AD** | **PH** | **GY** | **PA** | **EA** |
| --- | --- | --- | --- | --- | --- | --- | --- |
| 1 | 1 | -0.31 | -0.11 | 1.47 | 0.45 | -0.41** | -0.47** |
| 3 | 1 | 0.7 | 0.77 | 8.5 | 0.83** | -0.09 | -0.21 |
| 4 | 1 | 1.6** | 1.27** | -11.39* | -0.35 | 0.21 | 0.11 |
| 5 | 1 | 1.42** | 1.43** | -3.56 | -0.06 | 0.01 | -0.01 |
| 6 | 1 | 0.69 | 0.55 | 3.38 | 0.31 | -0.05 | -0.35* |
| 7 | 1 | -1.1* | -1.12** | 11.23* | 1.18** | -0.4** | -0.63** |
| 8 | 1 | 0.79 | 0.69 | -5.64 | -0.28 | -0.06 | 0.21 |
| 11 | 1 | 2.78*** | 2.19** | -26.99** | -1.35** | 0.46** | 0.47** |
| 12 | 1 | -2.04*** | -1.69** | 31.34** | 2.13** | -0.76** | -1.16** |
| 15 | 1 | -0.57 | -0.3 | 12.67** | 0.5 | -0.47** | -0.41** |
| 16 | 1 | 1.46** | 1.28** | -8.89* | -0.34 | 0.11 | 0.06 |
| 21 | 3 | 0.56 | 0.56 | -10.38* | -0.38 | 0.23 | 0.06 |
| 30 | 3 | 0.74 | 0.62 | -6.04 | -0.6* | -0.04 | 0.27 |
| 34 | 2 | 0.9 | 0.71 | 13.9** | -0.31 | 0.16 | 0.08 |
| 37 | 2 | 0.01 | 0.03 | 2.1 | -0.23 | 0.43** | 0.13 |
| 38 | 2 | 1.08* | 0.99* | 4.31 | 0.27 | -0.19 | -0.28 |
| 40 | 2 | 0.41 | 0.69 | 10.45* | -0.22 | 0.1 | 0.06 |
| 41 | 2 | 0.56 | 0.44 | 12.8** | -0.45 | 0.09 | 0.13 |
| 43 | 2 | 0.53 | 0.79 | 11.34* | 0.09 | -0.03 | -0.24 |
| 45 | 2 | 1.46** | 1.21** | -0.21 | -0.05 | -0.04 | -0.09 |
| 51 | 4 | 0.01 | 0.01 | 4.46 | -0.17 | 0.45** | 0.12 |
| 53 | 4 | -0.87 | -0.49 | 6.63 | 0.31 | 0.12 | -0.16 |
| 55 | 4 | -0.72 | -0.89* | -2.24 | 0 | 0.12 | 0.1 |
| 59 | 4 | -0.96 | -0.53 | -0.48 | 0.55 | -0.34* | -0.25 |
| 60 | 4 | -0.31 | -0.38 | 1.6 | -0.5 | 0.31* | 0.15 |
| 62 | 4 | -1.52** | -1.24** | 0.41 | 0.46 | 0.17 | -0.16 |
| 63 | 4 | 0.57 | 0.47 | 3.95 | -0.66* | 0.11 | 0.21 |
| 65 | 5 | -0.52 | -0.98* | -2.02 | -0.37 | 0.11 | 0.19 |
| 66 | 5 | -1.31** | -1.19** | -7.5 | -0.06 | -0.09 | 0.09 |
| 67 | 5 | -1.17* | -1.26** | -1.44 | -0.29 | -0.13 | 0.06 |
| 68 | 5 | -1.38** | -0.99* | 4.85 | 0.34 | 0.16 | 0.03 |
| 69 | 5 | -1.37** | -1.18** | -2.35 | 0.17 | -0.13 | 0.08 |
| 70 | 5 | -0.97 | -0.98* | -1.22 | -0.29 | -0.03 | 0.16 |
| 71 | 5 | -0.65 | -0.66 | -2.86 | -0.08 | 0.12 | 0.17 |
| 72 | 5 | -1.39** | -1.6** | 0.32 | -0.18 | 0.08 | -0.04 |
| 73 | 5 | -0.97 | -0.93* | 2.46 | 0.06 | -0.05 | 0.11 |
| 74 | 5 | -1.41** | -1.21** | -1.23 | -0.03 | -0.02 | -0.04 |
| 75 | 5 | -1.31** | -1.32** | -3.44 | -0.06 | 0.24 | 0.16 |
| 76 | 5 | -0.68 | -0.96* | -3.91 | -0.36 | -0.14 | 0.13 |
| 77 | 5 | -1.52** | -1.33** | -4.2 | -0.24 | 0.11 | -0.02 |
| 78 | 5 | -0.69 | -1.04* | 3.83 | 0 | 0.18 | 0.16 |
| 79 | 5 | -1.24* | -1.18** | 1.63 | 0.05 | -0.19 | -0.02 |
| 80 | 5 | -0.94 | -0.87* | -7.37 | -0.19 | -0.06 | 0.24 |
|  |  | 0.50 | 0.42 | 4.47 | 0.30 | 0.15 | 0.15 |

*, Significant at P < 0.05; ** significant at *p<*0.01; AD, no. of days to anthesis; PH, plant height (in cm); GY, grain yield (in t/ha); EA, ear aspect (1 to 5 score); PA, plant aspect (1 to 5 score)

**Note:** only hybrids with significant SCA effects for at least one trait are shown.

**Supplementary Table 9.** Percent mid-parent heterosis (mPH%) estimates for 42 hybrids out of the 80 hybrids generated from 24 inbred lines in a factorial mating design

| **Hybrid** | **Set** | **Female** | **Male** | **Heterosis (%)** | | | | | | **PVA**  **(μg/g)** |
| --- | --- | --- | --- | --- | --- | --- | --- | --- | --- | --- |
|  |  |  |  | **Lut** | **Zea** | **βcryp** | **βcar** | **PVA** | **Tcar** |  |
| **3** | **1** | **3** | **8** | 51.56** | -6.81 | 31.85** | 51.58*** | 47.56*** | 17.17 | 5.82 |
| **4** | **1** | **4** | **8** | 4.73 | 22.13 | 14.1 | 18.87* | 16.03 | 10.96 | 4.83 |
| **7** | **1** | **3** | **7** | 36.05* | 0.19 | -17.77 | 7.62 | 2.47 | 4.73 | 4.81 |
| **8** | **1** | **4** | **7** | 25.8* | 19.89 | -29.41 | 13.83* | 7.55 | 15.18 | 5.29 |
| **9** | **1** | **1** | **6** | 74.03** | -1.58 | 0.11 | 10.79 | 7.84 | 7.77 | 5.03 |
| **11** | **1** | **3** | **6** | 37 | 17.22* | 2.83 | 10.65 | 8.83 | 14.7* | 4.53 |
| **12** | **1** | **4** | **6** | 12 | 26.35* | 3.88 | 49.94*** | 38.05*** | 21.55** | 6.06 |
| **13** | **1** | **1** | **5** | 113.76*** | 21.86 | 26.55 | -2.63 | 0.77 | 27.73* | 5.85 |
| **15** | **1** | **3** | **5** | 43.08 | 36.99** | 29.76* | 16.92* | 18.58** | 28.21** | 6.29 |
| **18** | **3** | **10** | **4** | 16.6 | 27.44* | -7.96 | 27.97** | 18.28* | 17.41* | 4.95 |
| **19** | **3** | **11** | **4** | 8.2 | 20.11 | -5.18 | 22.99* | 15.21 | 11.06 | 4.76 |
| **20** | **3** | **12** | **4** | 29.51** | 35.22** | -24.3** | 20.59* | 6.58 | 21.43** | 5.43 |
| **21** | **3** | **9** | **3** | 46.4** | 5.45 | -1.39 | 26.01 | 14.46 | 9.72 | 4.41 |
| **22** | **3** | **10** | **3** | 7.43 | 16.23 | 29.87*** | 35.12** | 32.9** | 14.07 | 5.27 |
| **23** | **3** | **11** | **3** | 20.26 | 7.31 | 45.4*** | 58.18*** | 53.41*** | 14.92* | 6.00 |
| **27** | **3** | **11** | **2** | -10.33 | -7.61 | 23.72** | 20.86 | 20.94* | -6.37 | 4.72 |
| **30** | **3** | **10** | **1** | 48.48* | 25.83* | 18.03* | 21.43* | 20.81* | 23.94** | 5.39 |
| **31** | **3** | **11** | **1** | 8.87 | 11.69 | 39.22*** | 23.05* | 26.24** | 10.55 | 5.57 |
| **33** | **2** | **5** | **16** | 37.66 | 39.57** | 84.45*** | -4.75 | 6.73 | 28.28** | 5.04 |
| **36** | **2** | **8** | **16** | -22.49 | -7.19 | 50.59** | 29.84* | 33.38** | -5.95 | 4.48 |
| **37** | **2** | **5** | **15** | 20.79 | 45.65** | 74.53*** | 7.65 | 18.33** | 29.58** | 6.53 |
| **38** | **2** | **6** | **15** | 16.94 | 6.67 | 21.38** | 23.32* | 21.56* | 7.1 | 5.32 |
| **40** | **2** | **8** | **15** | 19.74 | -4.65 | 25.85* | 21.59* | 22.2* | 4.86 | 5.07 |
| **41** | **2** | **5** | **14** | 10.45 | 32.85** | 17.75 | -10.23 | -7.24 | 14.63 | 5.08 |
| **44** | **2** | **8** | **14** | 45.17** | -24.82* | 10.28 | -4.64 | -2.11 | -4.56 | 4.03 |
| **45** | **2** | **5** | **13** | 21.85 | 15.65 | 42.8** | -12.77* | -4.85 | 10.07 | 5.44 |
| **53** | **4** | **17** | **11** | 1.5 | -0.54 | 21.71* | 29.44 | 25.52* | 1.3 | 3.51 |
| **54** | **4** | **18** | **11** | 38.89*** | 18.35* | 23.95* | 30.07 | 28.45* | 22.72** | 3.74 |
| **55** | **4** | **19** | **11** | -7.83 | 2.37 | 26.1* | 25.92 | 24.23* | 0.04 | 3.56 |
| **56** | **4** | **20** | **11** | 18.24 | 10.61 | 21.76** | 25.43* | 23.89** | 11.44 | 4.98 |
| **57** | **4** | **17** | **10** | 1.21 | 34.13** | 83*** | 35.69* | 48.13*** | 24.45* | 4.22 |
| **58** | **4** | **18** | **10** | 3.49 | 29.63** | 27.02** | 38.74* | 33.78** | 19.41* | 3.97 |
| **59** | **4** | **19** | **10** | 2.69 | 6.67 | 26.66* | 29.19 | 27.62* | 5.29 | 3.73 |
| **61** | **4** | **17** | **9** | 33.22* | 8.99 | -11.34 | 33.45 | 12.79 | 10.94 | 3.08 |
| **68** | **5** | **24** | **20** | 38.38* | 45.97** | 8.88 | 43.87** | 29.27* | 32.77** | 3.78 |
| **70** | **5** | **22** | **19** | 33.95* | 25.53 | 4.66 | 28.93 | 19.95 | 21.22 | 3.06 |
| **72** | **5** | **24** | **19** | -4.78 | 44.83** | 14.11 | 15.82 | 11.35 | 17.48 | 1.98 |
| **74** | **5** | **22** | **18** | 14.33 | 16.05 | 17.35 | 43.5* | 34.69* | 15.29 | 3.50 |
| **76** | **5** | **24** | **18** | 1.49 | 47.96** | 19.05 | 47.15 | 34.26 | 27.17* | 2.45 |
| **78** | **5** | **22** | **17** | 18.18 | 34.45* | 16.85 | 52.72** | 39.65** | 28.73* | 3.46 |
| **79** | **5** | **23** | **17** | -17.16* | 21.43 | 48.38* | 39.13* | 40.48* | 3.62 | 3.25 |
| **80** | **5** | **24** | **17** | 49.37* | 71.51*** | 23.8 | 42.12 | 31.33 | 53.55*** | 2.24 |

Lut = Lutein, Zea = Zeaxanthin, βcryp = β-cryptoxanthin, βcar = β-carotene, PVA = Provitamin, Tcar = Total carotenoid

**Note:** only crosses with significant negative or positive heterosis for at least one of the carotenoids are shown
